# A Cell-based Computational Modeling Approach for Developing Site-Directed Molecular Probes

**DOI:** 10.1371/journal.pcbi.1002378

**Published:** 2012-02-23

**Authors:** Jing-yu Yu, Nan Zheng, Gerta Mane, Kyoung Ah Min, Juan P. Hinestroza, Huaning Zhu, Kathleen A. Stringer, Gus R. Rosania

**Affiliations:** 1 Department of Pharmaceutical Sciences, University of Michigan College of Pharmacy, Ann Arbor, Michigan, United States of America; 2 Department of Clinical, Social and Administrative Sciences, University of Michigan College of Pharmacy, Ann Arbor, Michigan, United States of America; 3 Fiber Science Program, Cornell University, Ithaca, New York, United States of America; Johns Hopkins University, United States of America

## Abstract

Modeling the local absorption and retention patterns of membrane-permeant small molecules in a cellular context could facilitate development of site-directed chemical agents for bioimaging or therapeutic applications. Here, we present an integrative approach to this problem, combining *in silico* computational models, *in vitro* cell based assays and *in vivo* biodistribution studies. To target small molecule probes to the epithelial cells of the upper airways, a multiscale computational model of the lung was first used as a screening tool, *in silico*. Following virtual screening, cell monolayers differentiated on microfabricated pore arrays and multilayer cultures of primary human bronchial epithelial cells differentiated in an air-liquid interface were used to test the local absorption and intracellular retention patterns of selected probes, *in vitro*. Lastly, experiments involving visualization of bioimaging probe distribution in the lungs after local and systemic administration were used to test the relevance of computational models and cell-based assays, *in vivo*. The results of *in vivo* experiments were consistent with the results of *in silico* simulations, indicating that mitochondrial accumulation of membrane permeant, hydrophilic cations can be used to maximize local exposure and retention, specifically in the upper airways after intratracheal administration.

## Author Summary

We have developed an integrative, cell-based modeling approach to facilitate the design and discovery of chemical agents directed to specific sites of action within a living organism. Here, a computational, multiscale transport model of the lung was adapted to enable virtual screening of small molecules targeting the epithelial cells of the upper airways. In turn, the transport behaviors of selected candidate probes were evaluated to establish their degree of retention at a site of absorption, using computational simulations as well as two *in vitro* cell-based assay systems. Lastly, bioimaging experiments were performed to examine candidate molecules' distribution in the lungs of mice after local and systemic administration. Based on computational simulations, the higher mitochondrial density per unit absorption surface area is the key parameter determining the higher retention of small molecule hydrophilic cations in the upper airways, relative to lipophilic weak bases, specifically after intratracheal administration.

## Introduction

**Figure 1:**
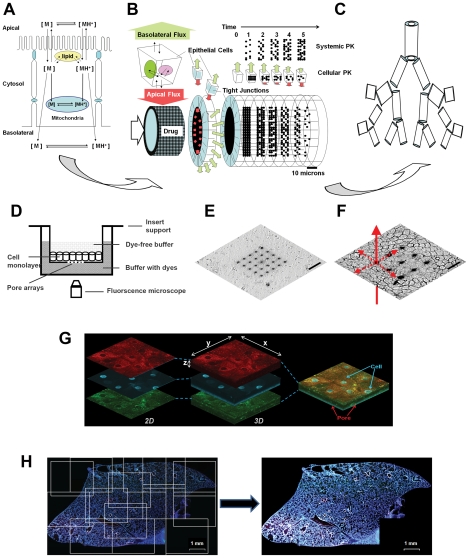
General methodology of integrative, cell based transport modeling. **A**) For computational simulations at the cellular level, a monobasic compound diffuses across a phospholipid bilayer and undergoes ionization and partition/binding in each compartment. The neutral form of the monobasic molecule is indicated as [M], and the protonated, cationic form of the molecule is indicated as [MH^+^]. **B**) For computational simulations at the histological level, each airway generation is modeled as a tube lined by epithelial cells; as molecules are absorbed over time, the drug concentration in the lumen decreases accompanied by an increase in drug concentration in the circulation **C**) For computational simulations at the organ level, the lung is modeled as a branching tree, with airway generation modeled as a cylinder, from the trachea to the alveoli. **D**) Experimental design of insert system with patterned pore arrays on membrane support for viewing lateral transport of fluorescent molecules along the plane of a cell monolayer, away from a point source. **E**) Transmitted light image of a 5×5, 3 µm diameter pore array (20 µm spacing) on a polyester membrane. **F**) Transmitted light image of an MDCK cell monolayer above a membrane support with 3×3, 3 µm diameter pore array (40 µm spacing). Scale bar: 40 µm. **G**) 3D reconstruction of confocal images of the distribution of three fluorescent probes added to the uppermost surface of NHBE cell multilayers grown on air-liquid interface cultures on porous membrane support. Each 3D plane is composed of the image with the fluorescent channel; red (MTR), blue (Hoe), and green (LTG). **H**) Illustration of the tiling algorithm used to visualize and quantify the distribution of Hoe and MTR in lung cryosections, after IT and IV coadministration of the probes.

Local administration of therapeutic agents or bioimaging probes is commonly used to maximize concentrations at a desired site of action and to minimize side effects or background signals associated with distribution in off-target sites. However, in the specific case of inhaled, small molecule therapeutic agents or bioimaging probes, cell impermeant molecules may rapidly disappear from the sites of deposition via mucociliary clearance [1], [2]. Conversely, cell- permeant small molecules can rapidly diffuse away and disappear from the site absorption, down their concentration gradient [3]. Therefore, we decided to explore an integrative simulation approach (Figure 1) to study how the physicochemical properties of small molecule probes may be optimized to maximize local targeting and retention in the upper respiratory tract.

Previously, we constructed multiscale, cell-based computational models of airways and alveoli to predict the relative absorption, accumulation and retention of inhaled chemical agents [4]. In these models, the transport of small molecules from the airway surface lining to the blood or from the blood to the airway surface lining were modeled using ordinary differential equations (ODEs) [5], [6]. These ODEs described the transport of drug molecules across a series of cellular compartments bounded by lipid bilayers (Figure 1A,), which form the surface of each airway generation, modeled as a tube (Figure 1B). For a monoprotic base, the concentration of molecule in each subcellular compartment was divided into two components: neutral and ionized [7], [8]. Accordingly, two drug specific properties were used as input to simulate the transport process across each lipid bilayer: the logarithms of the octanol∶water partition coefficient of the neutral form of the molecule (i.e., log*P_n_*) and the pK_a_ of the molecule. The logarithm of the octanol∶water partition coefficient of the ionized form of the molecule (i.e., log*P_d_*) can be derived from log*P_n_* or it can be incorporated as an independent input parameter that can be measured or calculated with cheminformatics software. For different compartments with different pHs and lipid fractions, the free fraction of the neutral and ionized forms of molecules was calculated according to the molecule's pK_a_, log*P_n_*, and log*P_d_*, using the Henderson-Hasselback equation and the laws of mass action.

Anatomically, the structure of the airways was modeled as a tree-like branching system of cylinders with progressively narrowing diameter [9] (Figure 1C). Starting with the trachea as the trunk of the tree and ending in the alveoli as the leaves, each branching segment corresponded to an airway ‘generation’ characterized by a particular surface area, blood flow, and cellular organization [4],[10]. Histologically, the walls of the airways or alveoli were modeled as multiple layers of epithelial, interstitial and endothelial cells separating the air from the blood. Several structural and functional differences between the airways and alveoli are noteworthy: 1) cartilage and smooth muscle are present only in the interstitium of the airways; 2) the surface area of the alveoli is two orders of magnitude larger than airways; and 3) while the blood flow to the alveoli corresponds to 100% of cardiac output from the right ventricle, the blood flow of the airways is approximately 1% of the cardiac output from the left ventricle [11], [12].

To predict a molecule's absorption and retention in each airway generation, the transport properties of small molecules across cellular membranes, as well as the local partitioning of molecules into lipid in different subcellular compartments can be calculated with the Fick and Nernst-Planck equations to describe the transport of the neutral and charged species of the molecule [4]. In simulations, combinations of logP and pK_a_ spanning a range of values were used as input to simulate the changes in concentration of molecules of varying chemical structure, as they are absorbed from the airway surface lining liquid into the blood or vice versa.

Here, we applied this cell-based transport model as a virtual screening tool, to identify compounds with differential distribution profiles in airways and alveoli, after intratracheal (IT) or intravenous (IV) administration. In addition, two innovative *in vitro* cell based assays were developed to assess the absorption and retention of molecules across multiple layers of cells along the lateral (Figure 1 D–F) and transversal planes of a cell monolayer (Figure 1G)). Finally, *in vivo* microscopic bioimaging experiments were performed to visualize the distribution of fluorescent probes in the lung after either IT or IV administration (Figure 1H). The results revealed that the mitochondrial sequestration of hydrophilic, cell-permeant cations can provide an effective mechanism for maximizing their local exposure and retention at a site of absorption. Accordingly, mitochondriotropic cations may be useful as fiduciary markers of local, inhaled drug deposition patterns in the upper respiratory tract.

## Methods

### General methodology

All of the equations and default parameter values were based on our published model [4]. The ODEs that describe this lung pharmacokinetic (PK) model were solved numerically in a Matlab® simulation environment (Version R2009b, The Mathworks Inc, Natick, MA). The ODE15S solver was used to address the issue of the stiffness in ODEs, and the relative and absolute error tolerance was set as 10^−12^ to minimize numerical errors. The Matlab scripts used for virtual screening and simulation purposes are provided, together with detailed instructions for running them, in the Supplementary Materials (Text S1, S2, S3, 4, S5, S6). The results of detailed parameter sensitivity analysis are also provided in the Supplementary Materials (Text S7).

### Virtual screening of small monobasic molecules targeting the airways after IT instillation

For virtual screening, the airway and alveoli were linked to a systemic pharmacokinetic model through their respective blood compartments using a single compartment PK elimination model (eq. 1) [13]:
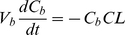
(1)Where *V_b_* is the volume of the blood compartment; *C_b_* is the concentration in the blood; and *CL* is the clearance. The same initial dose (1 mg/kg) was used as an input parameter to simulate IT instillation experiments in the airways and alveoli, respectively. For virtual screening, clearance in the systemic circulation was set to zero. The log*P_n_* (−2 to 4 with interval of 0.1 units) and the pK_a_ (5 to 14 with interval of 0.2 units) of monobasic compounds were independently varied and used as input parameters, in all possible combinations. For each set of physicochemical input parameters (log*P_n_* and pK_a_) two important pharmacokinetic indexes were calculated: 1) the percentage of mass deposited in the airways and alveoli (relative to the total mass in whole lung); and, 2) the concentration in the alveolar and airway regions, calculated as the sum of the masses in all the compartments in said regions of the lung divided by the sum of all the compartment volumes in that region. The area under the tissue concentration curve (AUC) for the airways and alveoli was calculated using the trapezoidal rule. The AUC ratio of airways to alveoli after inhalation was calculated by dividing the AUC of the airways by the AUC of the alveoli for every combination of log*P_n_* and pK_a_ that were used as input.

For comparison, simulations were also run to simulate an intravenous (IV) bolus injection, with the initial concentration in venous blood as calculated with eq. 2:
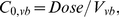
(2)The volume of venous and artery blood was set to 13.6 and 6.8 ml, respectively [13], [14]. The concentration in the blood was fixed (clearance set to 0) with the assumption of no significant plasma protein binding and a drug concentration blood to plasma ratio of 1.

### Detailed simulation analysis of fluorescent probe distribution in the airways and alveoli after local and systemic administration

Based on the results of virtual screening, two fluorescent probes were selected for further testing: Hoechst® 33342 (Hoe, Molecular Probes, CA, USA) to represent a highly hydrophobic, weakly basic molecule that can serve as a reference marker for a readily absorbed probe with limited intracellular retention; and, Mitotracker® Red (MTR, Molecular Probes, CA, USA) to represent a more hydrophilic cation that could serve as a candidate fiduciary marker for local inhaled drug deposition and absorption patterns. MTR was modeled with a single, fixed positive charge and a logP*_d_* = 0.16. Hoe was modeled as a lipophilic, monobasic molecule with a pKa = 7.8 and a logP*_n_* = 4.49 (calculated with ChemAxon, www.chemaxon.com). These physicochemical properties were used as input parameters to calculate the time dependent changes of the probe concentrations in the airways and alveoli, respectively. For simulations of IT instillation, the same initial concentration (1 mM) of MTR and Hoe was assumed as the initial condition for the airways and alveoli. The same initial dose used for IT instillation was also used for IV administration. Blood clearance was fixed to 0 for simulations, unless otherwise noted.

### Cell–based transport assays on microfabricated pore arrays

A customized transwell insert system was constructed using a polyester membrane with microfabricated pore arrays precisely machined using a focused ion beam (Hitachi FB-200A) [15] (Figure 1 E). These membranes support cell growth and the pores serve as a point source for compound administration to single cells on a cell monolayer (Figure 1F)). The pore arrays were comprised of 3 µm diameter cylindrical pores, arranged 20 µm apart in a 5-by-5 square array. Pores were also arranged 40, 80 and 160 µm apart in 3-by-3 symmetrical arrays. The pores were individually machined using a high brightness Ga liquid metal ion source coupled with a double lens focusing system. The perforated membranes were glued (Krazy Glue®) to the bottom of hollow Transwell® holder (Costar 3462 or 3460), creating a permeable support for cell growth (Figure 1D). The integrity of the insert system was tested by adding 5 mM Trypan Blue (dissolved in Hank's balanced salt solution; HBSS) to the insert wells [15]. The insert was considered intact if there was no evidence of Trypan Blue leakage from the edge of the insert membrane. For assessing lateral cell-cell transport, Madin-Darby canine kidney (MDCK) cells were purchased from ATCC (CCL-34™) and grown (37°C, 5% CO_2_) in Dulbecco's modified Eagle's medium (DMEM, Gibco 11995) containing 10% FBS (Gibco 10082), 1× non-essential amino acids (Gibco 11140) and 1% penicillin/streptomycin (Gibco 15140). MDCK cells were seeded on polyester membranes containing the pore arrays at a density between 1×10^5^–2×10^5^ cells/cm^2^ and were grown until a confluent cell monolayer formed (Figure 1F). To evaluate the effect of pore arrays on cell monolayer intactness, MDCK cells were washed and incubated in transport buffer (HBSS buffer supplemented with 25 mM D-glucose, pH 7.4) for 30 min followed by transepithelial electrical resistance (TEER) measurement using Millipore Millicell® ERS. Cell monolayers were used for experiments only if the background subtracted TEER values were higher than 100 Ω·cm^2^ and if the cells covering the pore arrays appeared as an intact monolayer.

### Measurement of lateral cell-to-cell transport and retention using microfabricated pore arrays

To assess cell-to-cell transport along the plane of the monolayer (Figure 1D–F), fluorescent dyes were added into the basolateral compartment of the transwell system (at time 0). The dynamic staining pattern in the cells was imaged (Nikon TE2000S epifluorescence microscope equipped with a triple-pass DAPI/FITC/TRITC filter set (Chroma Technology Corp. 86013v2)). The 12-bit grayscale images were acquired using a CCD camera (Roper Scientific, Tucson, AZ). For measurements, individual cells or nuclei in these images were manually outlined using the region tool in MetaMorph® software (Molecular Devices Corporation, Sunnyvale, CA). The average and standard deviation of cellular or nucleus fluorescence intensity was measured using MetaMorph®, after subtracting the background fluorescence intensity estimated from the unstained regions of the monolayer distant to the pores. The rate of Hoe staining in the nucleus was measured as the slope of fluorescence increase normalized by the slope of increase in the first nucleus (closest to the pore).

### Measurement of intracellular retention using Normal Human Bronchial Epithelial cell multilayers differentiated on air-liquid interfaces

Normal human bronchial epithelial (NHBE) cells (Clonetics™, passage 1; Lonza, Walkersville, MD) were cultured (37°C, 5% CO_2_) and seeded (passage 2) at 2.5×10^5^ cells/cm^2^ on a Transwell® insert (Corning Inc., Lowell, MA; area: 0.33 cm^2^, pore size: 0.4 µm) in NHBE differentiation media (Lonza, Walkersville, MD) The apical media was aspirated after 24 h of cell seeding and the cells on the polyester membrane were maintained in media only in the basolateral compartment of the air-liquid interface culture (ALC) [16], [17]. On day 8 of ALC, the integrity of the cell layers on the membrane was assessed by light contrast microscope and by transepithelial electrical resistance (TEER) [18]. After equilibration of the cell layers on the insert with pre-warmed HBSS buffer (10 mM HEPES, 25 mM D-glucose, pH 7.4) for 30 min (37°C, 5% CO_2_), TEER values were obtained and cells with TEER values of ∼600 Ω•cm^2^ were used for the transport and retention assays [16], [17], [19], [20], [21].

NHBE cell multilayers grown on the inserts were examined with a Zeiss LSM 510-META laser scanning confocal microscope (Carl Zeiss Inc., Thornwood, NJ) with a 60× water immersion objective on day 8 of ALC culture. For the confocal analyses, three different cell-permeant dyes were prepared by dilution with HBSS buffer 10 µg/ml Hoe; 2.5 µM LysoTracker® Green (LTG, Molecular Probes, CA); and 1 µM MTR). After the cell multilayers were washed with HBSS, 240 µl of dye mixture (80 µl of each dye in HBSS) was added to the apical compartment and 600 µl of HBSS was added to the basolateral side. After 30 min, transport of the dyes across the cell layers was measured by placing the insert into a two-chambered slide (Lab-TeK®; Thermo Scientific Nunc co., Rochester, NY) and acquiring images along the Z-axis (interval, 1 µm) in three fluorescence channels (coherent enterprise laser (364 nm) for Hoe, Argon laser (488 nm) for LTG, and Helium neon 1 laser (543 nm) for MTR). The distribution of probes applied in the apical compartment of the NHBE cell multilayer cultures was assessed in 3D reconstructions of the acquired images of probe distribution, using MetaMorph® software (Figure 1G). The relative distributions of MTR, Hoe, and LTG dyes across the multilayers were assessed by imaging analyses through multiple Z-stacks. After background subtraction, the integrated intensity of each fluorescence channel per cell was summed in each cell layer and divided by the total integrated intensity in all the layers to calculate the percentage of relative distribution of the integrated fluorescence signal of each dye associated with inner layer or the exposed surface layer of the NHBE cell multilayer.

### Visualizing probe distribution in mice lungs after IT instillation or IV administration

The distribution of MTR and Hoe in airways and alveoli after IT and IV injection in live mice were determined by microscopic imaging of cryopreserved lung tissue sections and confirmed by visual inspection followed by quantitative imaging of high resolution tiled mosaics assembled from fluorescence images of tissue sections (Figure 1H). For these experiments, male C57BL/6J mice (Jackson Laboratory, Bar Harbor, ME; 8 weeks, 20–30 g) were used and the protocol was approved by the University of Michigan's animal care and use committee in accordance with the National Institutes of Health Office of Laboratory Animal Welfare “Principles of Laboratory Animal Care.” MTR (50 ug in 10 ul DMSO) and Hoe (90 ul of 10 mg/ml in ddH_2_0) were mixed so that the final concentration of MTR and Hoe was 0.94 and 14.61 mM, respectively. Mice received either 50 µl of dye mixture or 50 µl saline (control) via IV tail veil injection or IT instillation [22]. For IV administration, conscious mice were briefly restrained and for IT instillation mice were anesthetized with isoflurane gas, and the dose was delivered to the airway via the oral route as previously described.

In order to study the differential regional distribution of fluorescent dyes in the lung, mice were anesthetized with ketamine/xylazine 40 minutes after dosing. A thoracotomy was performed and a heparinized blood sample was acquired by cardiac puncture. The trachea was cannulated (20G luer stub) after which the lungs were inflated with ∼1 mL of a 30% sucrose-optimal cutting temperature (OCT; Tissue-Tek, Sakura Finetek USA, Torrance, CA USA) mixture and removed *en bloc*. The lungs were immersed in OCT and were immediately frozen (at −80°C) [23].

For microscopy, coronal lung sections (7 µm) were imaged using an epifluorescence Olympus BX-51 microscope equipped with the standard DAPI, FITC and TRITC filter sets. A series of low-magnification (×4) left and right lung section images were electronically captured with an Olympus DP-70 high-resolution digital camera using Image J software (ImageJ 1.44b, National Institutes of Health, USA; http://rsb.info.nih.gov/ij). In order to permit comparisons of image brightness and fluorescence, images for each lung section were acquired using the same illumination and image acquisition settings. Mosaics of the entire lung were tiled using Photoshop® (version 4; Adobe Systems Inc., San Jose, CA) and quantitative image analysis was carried out using the integrated morphometric analysis function of MetaMorph®. Background subtracted fluorescence intensity values over the airways and alveoli were measured, as the integrated value of all pixels per unit area of the manually selected airway and alveolar tissue regions, using the images acquired with the DAPI channel. In turn, the same airway and alveolar tissue regions were used to measure the MTR fluorescence signal using the images acquired with the TRITC channel.

## Results

**Figure 2:**
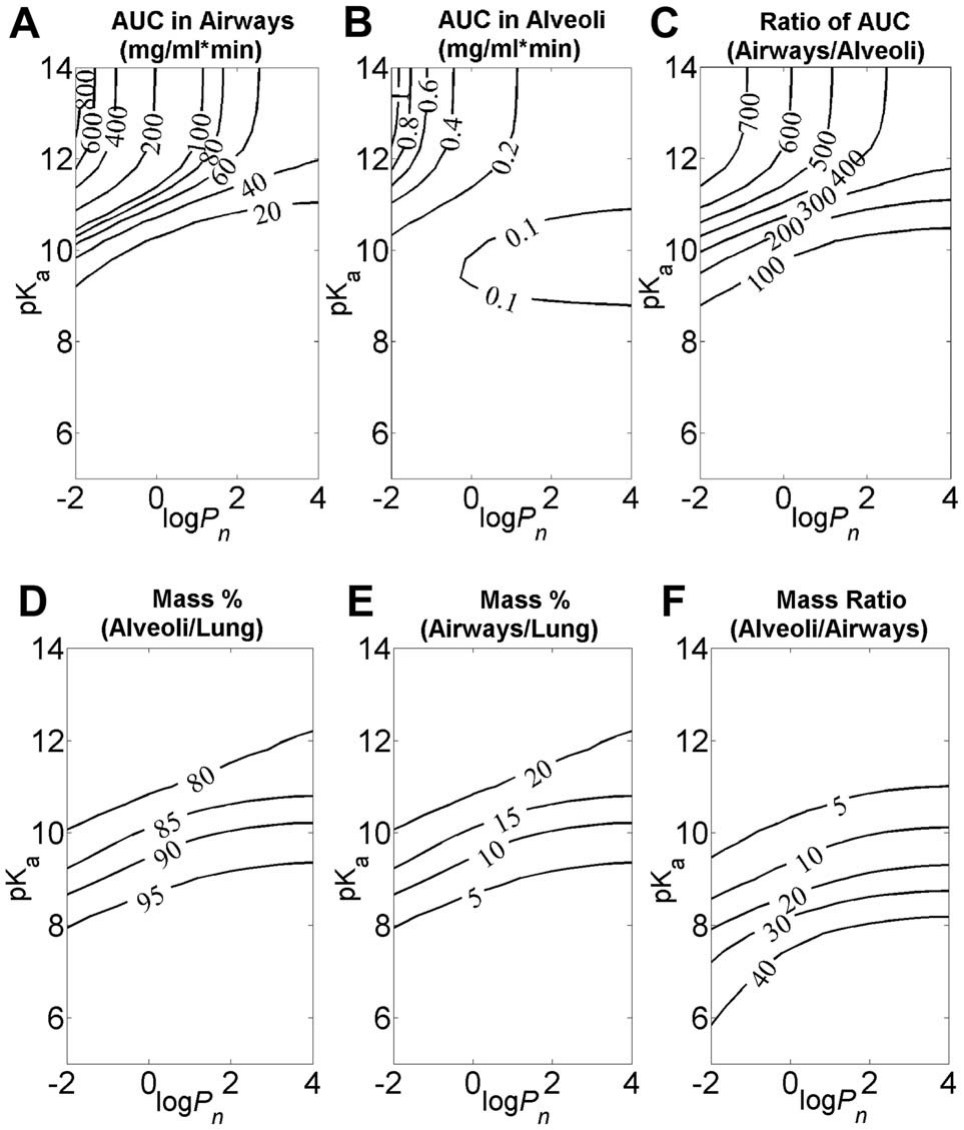
Virtual screening of monobasic compounds based differential tissue distribution in the airways and alveoli. The combinations of log*P_n_* and pK_a_ were used as input. For simulations, the initial dose was set to 1 mg/kg for airways and alveoli. Contour lines indicate: **A**) The calculated AUC (unit: mg/ml*min) in airways; **B**) The AUC (unit: mg/ml*min) in alveoli; **C**) The AUC contrast ratio of airways to alveoli; **D**) The mass percentage (%) in alveoli relative to the total mass in lung; **E**) The mass percentage (%) in airways relative to the total mass in lung; **F**) The mass ratio of alveoli to airway. Matlab scripts used to generate plots A–C (Text S1) and D–F (Text S2) are included in the supplementary materials.

For virtual screening experiments, molecules with maximal tissue exposure (AUC) in the airways after inhalation were identified by using combinations of log*P_n_* and pK_a_ as input parameters in a multiscale, cell-based lung transport model (Figure 2). For weak bases, lower lipophilicity and higher pKa promoted intracellular retention and led to greater local exposure relative to the alveoli (Figure 2A, B). The calculated airway/alveoli exposure ratio (Figure 2C) ranged from 100 to 700 and increased with lowered log*P_n_* (increasing hydrophilicity) and higher pK_a_ (greater positively charged fraction at physiological pH) Essentially, cell-permeant, hydrophilic molecules harboring a fixed positive charge showed the greatest accumulation and retention in the cells of the upper airway relative to the alveoli, following IT administration.

To probe the role of the route of administration, simulations were also performed by independently varying log*P_n_* and pK_a_ to calculate the mass deposition pattern in the airways and alveoli under steady state conditions after IV administration (Figure 2D–F). In this manner we established the relationship between the physicochemical properties of small molecules and absolute and relative mass distribution in the airways (Figure 2D) and alveoli (Figure 2E). Following IV administration, the majority of the mass was deposited in the alveoli irrespective of the physicochemical properties of the molecules (Figure 2F); the airways held less than 20% of total drug mass in the lungs. Compounds with low log*P_n_* and high pK_a_ tended to exhibit the largest airway to alveoli mass ratios, which paralleled the results obtained after IT administration.

**Figure 3:**
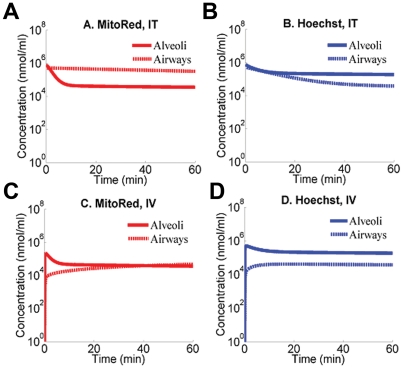
Simulations of local pharmacokinetics of MTR and Hoe after IV an IT administration. **A**) The simulated tissue concentration in airways (dash line) and alveoli (solid line) of MTR administered by IT instillation (Matlab script used to generate this plot is included as Text S3 in the supplementary materials); **B**) The simulated tissue concentration in airways (dash line) and alveoli (solid line) of Hoe administered by IT instillation (Matlab script used to generate this plot is included as Text S4 in the supplementary materials); **C**) The simulated tissue concentration in airways (dash line) and alveoli (solid line) of MTR administered by IV injection (Matlab script used to generate this plot is included as Text S5 in the supplementary materials); **D**) The simulated tissue concentration in airways (dash line) and alveoli (solid line) of Hoe administered by IV injection (Matlab script used to generate this plot is included as Text S6 in the supplementary materials).

In order to validate the results of these virtual screening experiments, two fluorescent bioimaging probes, MTR and Hoe, were selected for more detailed analysis. MTR is a cell-permeant, hydrophilic cation, and Hoe is a cell permeant, hydrophobic weak base. Based on the screening results (Figure 3) and more detailed simulations (Figure 3), the concentration profiles of these two fluorescent molecules in the airways and alveoli were markedly different after IT (Figure 3 A, B) and more similar after IV (Figure 3 C, D) administration. When given IT, the predicted MTR concentration, 40 to 60 min after administration, was nearly 10-fold higher in the airways than in the alveoli (Figure 3A). Conversely, the predicted concentration of Hoe in the airways was two-fold higher in alveoli than in airway (Figure 3B). When given IV, the predicted concentration of MTR in the airways was almost the same as that in alveoli (Figure 3C). However, the predicted concentration of Hoe in the airways was higher in alveoli (Figure 3D). Thus, MTR should be retained in the airways specifically after IT administration, whereas Hoe should not be retained in airways relative to alveoli regardless of the route of administration.

Next, cell based assays were used to establish the intracellular retention of MTR and Hoe at a site of absorption. For this purpose, a transwell insert system with micro-fabricated pores was constructed. After seeding MDCK epithelial cells on the patterned pore arrays and adding hydrophobic fluorescent compounds in the basolateral side of cell monolayer, the time course dye uptake in the cells sitting above the pores and the kinetics of lateral transport from the cells lying on top of the pore to the neighboring cells was visualized by fluorescence microscopy.

**Figure 4:**
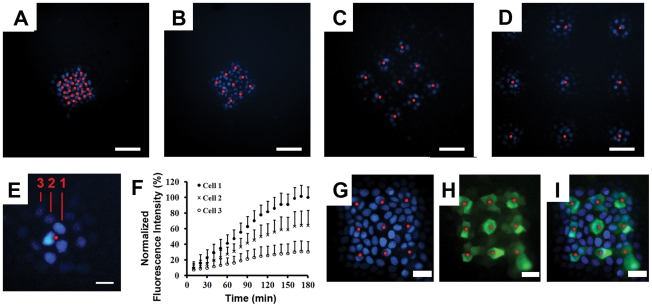
Probing the intracellular retention of Hoe along the plane of a cell monolayer. For the experiments, Hoe was added to the basolateral compartment and incubated for 3 hrs, with cell monolayers sitting on top of patterned pore arrays. Red spots indicate the location of pores; Scale bar: 80 µm. Cells were imaged using the DAPI channel of an epifluorescence microscope. **A**) 5×5 array of 3 µm pores with 20 µm spacing; **B**) 3×3 array of 3 µm pores with 40 µm spacing; **C**) 3×3 array of 3 µm pores with 80 µm spacing; **D**) 3×3 array of 3 µm pore array with 160 µm spacing; **E**) Fluorescent images of a cell monolayer incubated for 3 hours in the presence of Hoe in the basolateral compartment; **F**) corresponding measurements of fluorescence intensity of cells in A), showing the average fluorescence of each nucleus normalized by the average fluorescence of the nucleus closest to the pore at the 3 hr time point, and plotted as mean ± s.d. (n = 6). **G**) Fluorescence image of cell monolayer on a 3×3 array of 3 µm pores with 40 µm spacing after 2 hr incubation with Hoe and BCECF-AM in the basolateral compartment; **H**) FITC channel corresponding to BCECF staining of the same cells as in; **I**) Image overlays of C and D.

Three hours after the addition of Hoe to the basolateral compartment, only cells that were within close vicinity of pores were stained, indicating that the cells formed a tight seal with the pores such that each pore fed almost exclusively into cells that were in immediate contact with the pores (Figure 4). Monitoring of the cell-to-cell diffusion of Hoe over time, indicated that the pores served as point sources of sustained dye supply to the adjacent cells (Figure 4A–D) and for cells grown on membranes with pores spaced by 80 µm (Figure 4C) or 160 µm (Figure 4D), each pore could be considered as the single point source of dye molecules. Quantitative image analysis revealed that the rate of staining rapidly decreased as the distance of the cells from the pores increased (Figure 4E, F). Remarkably, only cells in the vicinity of each pore were labeled.

As controls, cells were stained with Hoe plus BCECF-AM from the basolateral compartment (Figure 4G–I). BCECF-AM is a nonfluorescent cell-permeant ester, which generates a cell-impermeant, fluorescent molecule upon intracellular hydrolysis. While the extent of Hoe diffusion was dependent on the distance from the pores (Figure 4G), the green fluorescence of the hydrophillic ester hydrolysis product (BCECF) was exclusively restricted to the first layer of cells that were in direct contact with pores (Figure 4H, I).

**Figure 5:**
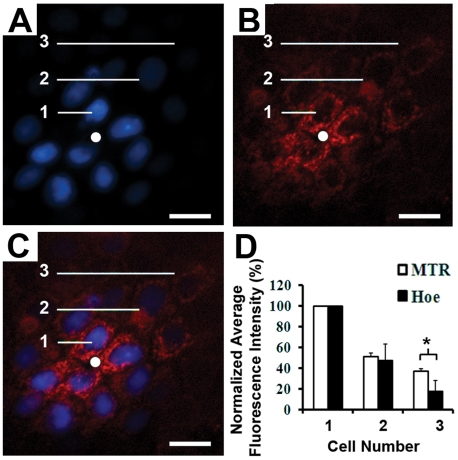
Probing the intracellular retention of MTR along the plane of a cell monolayer. Cell monolayers on pore arrays were incubated for 2 hr with Hoe and MTR in the basolateral compartment. White spots indicate the location of pores; Scale bar: 20 µm. **A**) Fluorescent image acquired with the DAPI channel showing Hoe diffusing on a cell monolayer sitting on top of a single pore of a 3×3 array of 3 µm pores with 160 µm spacing; **B**) Same field as in A, visualized with the TRITC channel to show the staining of MTR; **C**) Overlay of A and B showing the overlapping Hoe (blue) and MTR (red) staining patterns. **D**) Plots of the fluorescence intensity of Hoe and MTR, separated by 0, 1, 2 or 3 layers of cells from a pore, and normalized by the fluorescence intensity of the cell closest to the pore; asterisk indicates a statistically significant difference using Student's T-test; p<0.05; n = 6).

Similar to the Hoe staining pattern, MTR also exhibited a highly constrained diffusion pattern with most of the staining restricted to the vicinity of each pore (Figure 5). After two-hours of staining from the basolateral compartment with both Hoe (Figure 5A) and MTR (Figure 5B), only cells within 60 microns of the pore being stained with both probes (Figure 5C). The normalized fluorescence intensity of MTR and Hoe were similar in the first and second layers of cells, but MTR showed higher penetration into the third layer (Figure 5D).

**Figure 6:**
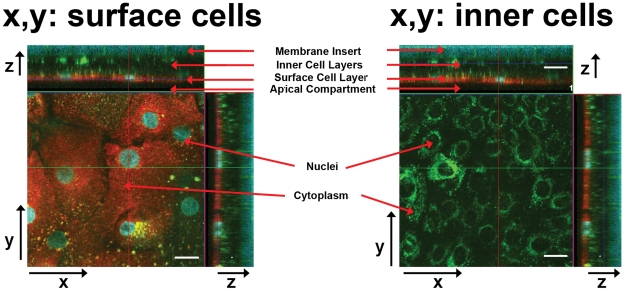
Fluorescent confocal images of NHBE cell multilayers on the porous membrane with Z-stacks. Cell multilayers were stained with MTR, Hoe and LTG. Each compartment (membrane inserts (bottom), inner cell layers, surface cell layer, and apical compartment (top)) through z-axis were indicated with the red arrows in x–z planes while cell nuclei and cytoplasm in x–y planes. The panel to the left shows an x, y cross section through the apical surface layer of the cell multilayer. The panel to the right shows an x, y cross section through the inner cell layer of the cell multilayer. Scale bar: 20 µm.

In the transversal direction, the absorption and retention of MTR and Hoe across multiple layers of cells was also assessed in primary NHBE cells differentiated as multilayers in ALC (Figure 6). For the experiments, MTR and Hoe were simultaneously added in the apical side of the cells and intracellular accumulation was assessed using 3D reconstructions of the cell multilayers (Figure 6). As a positive control, LTG was also included in the apical HBSS buffer. Thirty minutes after the addition of probes to the apical compartment, both MTR and Hoe staining were constrained to the first, outer surface layer of cells (Figure 6, left). The cells beneath the surface layer of cells were stained with LTG (Figure 6, right), indicating that the limited penetration of both MTR and Hoe. Different transport patterns of MTR, Hoe and LTG across the cell multilayers were verified by image quantitation using MetaMorph® software in the multiple Z-stack images of NHBE cell multilayers. Approximately 96%±2.76% of MTR or 96%±2.48% Hoe of the dye was retained in the surface cell layer whereas 50%±15.62% of LTG fluorescence was associated with the surface cell layer. Tukey's multiple comparison test following ANOVA (one-way analysis of variance) test showed statistically significant difference between MTR and LTG (p-value<0.0001) and also between Hoe and LTG (p-value<0.0001), but not between MTR and Hoe with p-value larger than 0.05 (α = 0.05).

**Figure 7:**
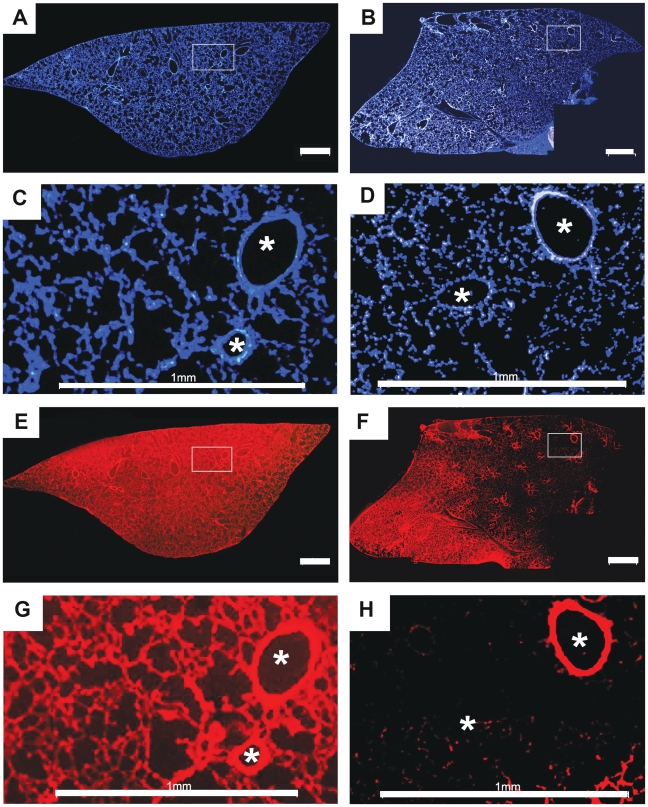
Tiled fluorescent micrographs of coronal cryosections obtained from the left lungs of mice. Mice received either an IV (A, C, E, G) or IT (B, D, F, H) dose of a mixture of Hoe and MTR. **A**) DAPI channel fluorescence image showing Hoe distribution following IV administration; **B**) DAPI channel fluorescence image showing Hoe distribution following IT administration; **C**) High magnification view of the boxed region in A; **D**) High magnification view of the boxed region in B; **E**) TRITC channel fluorescence image showing MTR distribution following IV administration; **F**) TRITC channel fluorescence image showing MTR distribution following IT administration; **G**) High magnification view of the boxed region in E; **H**) High magnification view of the boxed region in F. Scale bar = 1 mm. Asterisks mark the cross-sections of the airways, apparent as ellipsoids at high magnification.

As an ultimate test of the results of *in silico* virtual screening experiments, mice were administered a mixture of MTR and Hoe by either IV tail vein or IT instillation and the distribution of the molecules in the lungs was assessed by fluorescent microscopy (Figure 7). Hoe distributed throughout the lungs regardless of route of administration (Figure 7A, B) with fluorescence in both alveoli and airways (Figure 7C, D)). Following IV administration, MTR also distributed throughout the lung in both airways and alveoli (Figure 7E). Conversely, IT administered MTR resulted in highly uneven fluorescence distribution (Figure 7F). Most importantly, the airway regions showed comparable MTR fluorescence in airway vs. alveoli after IV (Figure 7G) but higher MTR fluorescence intensity in airways compared with the alveoli following IT delivery (Figure 7H).

To confirm these observations quantitative image analysis was performed to compute background subtracted integrated intensity of alveolar and airway regions, to quantify the relative, differential fluorescence intensity distribution of Hoe and MTR in airway and alveoli. The fluorescence MTR/Hoe ratio ranged from 2.42 to 3.27 for IT administration. For MTR and Hoechst, the mean (± s.d.) percent airway delivery was 23.9%±5.8% and 8.8%±2.7%, respectively (based on 422 region measurements from a single lung). For IV administration, the fluorescence MTR/Hoe ratio ranged from 0.95 to 1.45. The mean (± s.d) percent airway delivery for MTR and Hoe were 7.5%±2.5% and 7.1%±1.8%, respectively (based on 383 region measurements from a single lung). The images and measurements were consistent with local intracellular retention of MTR in the airways compared with Hoe, following IT (but not IV) instillation. These *in vivo* results paralleled the *in silico* simulation results (Figure 3).

In order to identify the most important parameters that might explain the differences in local retention of MTR and Hoe, a parameter exchange analysis was performed using computational simulations. For this purpose, individual parameters of the airway were exchanged with those of the alveoli, one at a time, and the simulations were rerun to calculate the exposure (AUC) of MTR and Hoe. Based on the results of this simulation analysis (Table 1) the volume of interstitial smooth muscle cells together with the volume of mitochondria were the primary factors determining the retention of MTR in the upper airways relative to alveoli. Secondarily, the surface areas of epithelial and endothelial cell layers were important, affecting retention in opposite directions. Taken together, these results suggest that the mitochondrial density per unit absorption surface area is the key histological organization parameter responsible for the higher retention of MTR in upper airways after IT administration.

**Table 1:** Results of parameter exchange analysis.

	Airways	Alveoli
Epithelium Surface Area	+	−
Smooth Muscle Volume	−−−	++
Endothelium Area	−	+
Macrophage Volume	unaffected	unaffected
Immune Cells Volume	unaffected	unaffected
Interstitium Volume	−−	−
Mitochondria Volume	−−	+
Blood Flow Rate	unaffected	unaffected
Clearance	unaffected	−

Table indicates the direction in which the exposure (AUC) of MTR changes after IT instillation, upon exchanging the indicated parameter values between airway and alveoli. A plus indicates an increase, while a minus indicates a decrease in AUC relative to the baseline lung model parameters. One plus or minus sign corresponds to a 1.1 to 1.5 fold change in AUC; two plus or minus signs to a 1.5 to 2 fold change; and three plus or minus signs to a >5 fold change. For clearance, the parameter value was increased 10-fold.

## Discussion

In traditional pharmacokinetic studies, drug distribution in the lungs is analyzed in a homogeneous and well-stirred compartment [13], [24]. Here, we have elaborated an integrated, cell-based approach to model local drug absorption and transport phenomena, aimed at identifying cell-permeant molecules that are retained in the cells of the upper airway upon local pulmonary administration via the inhaled route. This integrated approach can be exploited for bioimaging probe development or for optimizing the local concentration of pulmonary medications [25], [26].

Locally acting, inhaled medications are of considerable interest for treating various pulmonary ailments, including asthma, chronic obstructive pulmonary disease (COPD) and pulmonary hypertension [11], [27], [28]. The therapeutic benefits of inhaled medications include targeted drug delivery, rapid onset of action, low systemic exposure with a resultant reduction in systemic side effects [29], . Nevertheless, measuring local drug concentrations in the lungs is challenging. Previously, regional differences in local lung exposure have received little attention in the context of small molecule targeting and delivery. Inhaled drug development efforts ignore the possibility that local differences in drug exposure could influence regional differences in drug transport properties that are associated with structural and functional characteristics of the airways and alveoli [31], [32], [33]. Accordingly, the approach presented here is significant because it furthers our understanding of how inhaled drug molecules and bioimaging probes behave after local administration to the lungs. These findings have important implications in pulmonary drug development.

Our simulations and experiments indicate that route of administration, histological organization and circulatory parameters can affect the retention and distribution of different molecular agents in various regions of the lung based on the lipophilicity and ionization properties of molecules, and as such, may be of pivotal importance for the optimization of drug targeting [25], [26]. Specifically, we considered two major and clearly distinguishable regions of the lungs: the airways and the alveoli, which are histologically and physiologically distinct. Extensive studies have demonstrated that the regional lung deposition of drugs is largely dependent on the aerodynamic particle size generated by delivery devices [28], [31], [34], [35]. Here, we introduce the concept that other parameters (e.g., the chemical properties of molecules) may be as important for predicting the behavior of pulmonary delivered of drugs. This is evidenced from our simulations which indicated that, after absorption into the blood, the majority of drug mass (>80% of total mass in lungs) is predicted to accumulate in the alveoli because of its larger volume and higher lipid content and compounds with high lipophilicity and low pK_a_ will accumulate to even a greater extent in the alveoli. Although inhaled drug targeting leads to most of the drug mass deposited in the upper airways, without significant intracellular retention, the molecules can be rapidly absorbed and circulate back to the lung to accumulate in the alveoli. In theory, only molecules that are retained in the cells of the upper airways at the local site of administration can be effectively targeted to the upper airways.

To study the transport properties of small molecules in airways and in alveoli, we conducted simulations concentrated on characterizing the behavior of two fluorescent compounds, MTR or Hoe, because they exhibited large differences in simulated transport behaviors. In addition, two *in vitro* cell based assays were developed to test the local cellular uptake and retention properties of small molecules: 1) primary NHBE cell cultures comprised of cell multilayers differentiated on transwell insets in the presence of an air-liquid interface; and 2) MDCK cell monolayer cultures on microfabricted pore arrays to establish the lateral cell-to-cell transport kinetics of small molecules, along the plane of the cell monolayer. In the case of Hoe and MTR, both *in vitro* assays confirmed that the probes were taken up and largely retained by cells in the immediate vicinity of site of absorption and that the extent of diffusion followed a dye concentration gradient from the pores. Our *in vitro* findings indicated that the lateral cell-to-cell diffusion of MTR and Hoe was highly constrained. These *in vitro* results confirmed that both Hoe and MTR were retained intracellularly at a significant level in the presence of a transcellular concentration gradient both in the apical-to-basolateral and lateral directions. These results were also informative in terms of the time scale of intracellular accumulation and the relative labeling intensity afforded by these two fluorescent probes in the presence of a transcellular gradient. However, the *in vitro* assays did not reveal a major difference in the local retention of MTR and Hoe. Based on this observation, the behavior of these probes in these *in vitro* assays appeared most consistent with the predicted behavior of the probes in the alveoli.

Nevertheless, the results of *in vivo* studies closely paralleled those obtained *in silico*, in that MTR was retained in airways upon local IT administration while Hoe distributed in both airways and alveoli irrespective of the route of administration. Although *in vitro* results were useful to confirm the high, local intracellular retention of the probes, the *in silico* model is a better representation of the three-dimensional organization and physiological parameters of the *in vivo* situation. Parameter sensitivity analysis indicates that mitochondrial uptake of hydrophilic cations, in relation to the surface area over which absorption occurs, is the critical histological component responsible for high exposure of MTR when given via IT instillation. This is because as MTR traverses from the lumen of the airway into the interstitium, it is rapidly taken up into the mitochondria, driven by the high negative membrane potential of the mitochondrial inner membrane. Conversely, release of MTR from the mitochondria out into the circulation is very slow because the membrane potential slows its release. In the case of alveoli, the alveolar epithelial cells have much higher apical and basolateral plasma membrane surface areas relative to the mitochondrial membrane surface area. The higher cell surface areas facilitate mass transport of MTR across the cells and into the circulation, which reduces MTR accumulation in mitochondria.

In contrast to MTR, Hoe is a lipophilic weakly basic compound with a pKa of 7.5. Therefore, at physiological pH, half of the Hoechst molecules exist in a highly membrane-permeant, neutral form. Transmembrane diffusion of the neutral form of Hoe is orders of magnitude faster than that of a cationic form. So there is no significant accumulation or retention of Hoe in either the airways or the alveoli. When administered by IV injection, the direction of distribution is from blood to the tissue. The distribution between blood and tissue is mostly a function of the partitioning or binding of molecules from the circulation to the tissue, which is dependent on the cell density of the tissue, the membrane content of the tissue, and the affinity of the probes for membranes and intracellular components in the tissue. Thus, after IV administration, both Hoe and MTR tended to partition more into alveoli than into the airways.

In conclusion, we have elaborated an integrated *in silico*-to-*in vitro*-to-*in vivo* modeling approach which has applicability toward the optimization of site-specific targeting of locally-administered molecules. In the process, we have found that MTR is a candidate fiduciary marker for local drug deposition and absorption patterns in the airways. Due to the compartmental nature of the lungs, computational simulations can be linked to upstream process, such as pulmonary particle deposition, dissolution and mucus clearance, as well as to downstream processes that can be captured by pharmacodynamic models [36], [37], [38]. With additional effort this approach can be expanded to include macromolecules, acidic, zwitterionic molecules as well as molecules possessing multiple ionization sites, to further development of probes of lung structure and function [39], [40].

## Supporting Information

Text S1 **Virtual screening and simulation scripts File AUC_inhale.pdf containing detailed instructions and Matlab code used to generate** **figure 2A–C** **.**
(PDF)

Text S2 **File AW_AL_Mass.pdf containing detailed instructions and Matlab code used to generate** **figure 2D–F** **.**
(PDF)

Text S3 **File MTR_Inhale.pdf containing detailed instructions and Matlab code used to generate** **figure 3A** **.**
(PDF)

Text S4 **File HOE_Inhale.pdf containing detailed instructions and Matlab code used to generate** **figure 3B** **.**
(PDF)

Text S5 **File MTR_IV.pdf containing detailed instructions and Matlab code used to generate** **figure 3C** **.**
(PDF)

Text S6 **File HOE_IV.pdf containing detailed instructions and Matlab code used to generate** **figure 3D** **.**
(PDF)

Text S7 **Supplementary parameter values and sensitivity analysis.** This file includes information detailing: 1) Constant input parameter values used in the simulations; 2) Parameter values and sensitivity analysis of AUC (mg/ml*min) for alveoli; 3) Parameter values and sensitivity analysis of AUC (mg/ml*min) for airways; 4) Parameter values and sensitivity analysis of the time to reach steady state (Tss, in min) for alveoli; 5) Parameter values and sensitivity analysis of the time to reach steady state (Tss, in min) for airways; 6) Parameter values and sensitivity analysis of mass deposition for alveoli as a fraction of the total mass in the lung; 7) Parameter values and sensitivity analysis of mass deposition in airways as a fraction of the total mass in the lung.
(PDF)
