# Supplementary material for: A Cell-based Computational Modeling Approach for Developing Site-Directed Molecular Probes
Source: PLoS Comput Biol. 2012 Feb 23;8(2):e1002378. doi: 10.1371/journal.pcbi.1002378 (PMC3285574; doi:10.1371/journal.pcbi.1002378)
Supplement: Text S6 — File HOE_IV.pdf containing detailed instructions and Matlab code used to generate figure 3D . (PDF) [file pcbi.1002378.s006.pdf]

Instruction
m code for Figure 3 -D

There are five m files in this PDF file. Their locations were listed in the table below. Put all five m files as noted below into same folder, and run Main.m file to run the simulation.

m file name	Pages (starting – end page)
H_aw_RL.m	2-13
H_al_RL.m	14-23
H_Lung_RL.m	24-31
H_RL_IH_fun.m	32-33
Main.m	34-34

```
 % 4/30/2009
% 1CellPK based lung:Airways model starts here (Rats)
% 12 compartments:
% aEp (surface lining liquied), imEp (Macrophage),
% cEp(epithelial cells),cEpMito(mito of cEp), cEpLyso (lyso of cEp)
% int(Interstitium),imInt(immune cells), sm(smooth muscle),
% cEd(endothelial cells), cEdMito(mito of cEd),cEdLyso (lyso of cEd), p(plasma)

function [M, G, M_v, Vp] = H_aw_RL()


%molecular physiochemical property

pKa = 7.87;
logPN = 4 ;
logPD = logPN-3.7 ;
z = 1;
% Constant
T = 273.15+37;
R = 8.314;
F = 96484.56;
%lipid fraction
LaEp = 0.2;
LimEp = 0.05;
LcEp = 0.05 ;
Lint = 0;
Lsm = 0.05;
LimInt = 0.05;
LcEd = 0.05;
Lp = 0;
%volumetric water fraction=1-lipid fraction
WaEp = 1 - LaEp;
WimEp = 1 - LimEp;
WcEp = 1 - LcEp;
Wint = 1 - Lint;
Wsm = 1 - Lsm;
WimInt = 1 - LimInt;
WcEd = 1 - LcEd;
Wp = 1 - Lp;
%activity coefficient of species(N:neutral,D:desociated)
GaEpN = 1;
GaEpD = 1;
GimEpN = 1.23;
GimEpD = 0.74;
GcEpN = 1.23;
GcEpD = 0.74;
GintN = 1;
GintD = 1;
GsmN = 1.23;
GsmD = 0.74;
GimIntN = 1.23;
GimIntD = 0.74;
GcEdN = 1.23;
GcEdD = 0.74;
GpN = 1;
GpD = 1;
```

```
% By Jingyu Yu (used in publication) parameters in airways
% Areas and volumes (m^2, m^3) for 7 membranes and corresponding compartments
AaEp = 108*10^(-4);%literature
AbEp = AaEp;%assuming same with apical
AimEp = 0;%No macrophage
AimInt = 0.01*AaEp;%estimate
Asm = AaEp*2; % two side, double the surface area of airway,T model
AbEd = AaEp/5;%estimated 1/5 surface of epithelium
AaEd = AbEd;% same as basical side
%volumes for 8 compartments(m3)
ASL = 15; %um literature
VaEp = AaEp*ASL*10^(-6); %15 um thickness
VimEp = 10^(-30); %10^(-12)*VaEp; % Anynumber,No macrophage at surface
VcEp = 0.072*10^(-6); % estimated from yori model,basement membrane->surface area->thickness of each
generation
Vint = AaEp*1*10^(-6);%estimated
Vsm = 0.047*10^(-6);%% estimated from yori model,basement membrane->surface area->thickness of each
generation
VimInt = 0.01*Vint;%setimated
VcEd = AbEd*0.4*10^(-6); %estimated from literature,thickness of endothelium in AW

R_org = 0.1;
% calculate constant
VcEpMito = R_org*VcEp;
VcEpLyso = R_org*VcEp;
VcEdMito = R_org*VcEd ;
VcEdLyso = R_org*VcEd ;
VsmMito = R_org*Vsm;
VsmLyso = R_org*Vsm;

AcEpMito = 5.9924e+006*VcEpMito ;
AcEpLyso = 5.9924e+006*VcEpLyso ;
AcEdMito = 5.9924e+006*VcEdMito ;
AcEdLyso = 5.9924e+006*VcEdLyso ;
AsmMito = 5.9924e+006*VsmMito ;
AsmLyso = 5.9924e+006*VsmMito ;
%#########################################################################

M_v = diag([VaEp,VimEp,VcEp,VcEpMito,VcEpLyso,Vint,Vsm, VsmMito, VsmLyso, VimInt, VcEd, VcEdMito,
VcEdLyso]);
V_LUN = trace(M_v)*10^6;
Vp = 340*10^(-9)*V_LUN;

% Membrane potential (V)
EaEp = -0.0093;
EbEp = 0.0119;%0.0119;
EimEp = -0.06;
Esm = -0.06;
EimInt = -0.06;
EbEd = -0.06;
EaEd = -0.06;
% pH values
pHaEp = 7.4;
pHimEp = 7.0;
pHcEp = 7.0;
```

```
pHint = 7.0;
pHsm = 7.0;
pHimInt = 7.0;
pHcEd = 7.0;
pHp = 7.4;


%adjustment for logP
if ( abs(z-1) <= 10^(-6) )
   logP_nlipT = 0.33*logPN+2.2 ;
   logP_dlipT = 0.37*logPD+2 ;
end
if ( abs(z+1) <= 10^(-6) )
   logP_nlipT = 0.37*logPN+2.2 ;
   logP_dlipT = 0.33*logPD+2.6 ;
end
if ( abs(z-0) <= 10^(-5) )
   logP_nlipT = 0.33*logPN+2.2 ;
   logP_dlipT = 0.33*logPD+2.2 ;
end

% Get the first two decimals
logP_n = round(logP_nlipT*100)/100 ;
logP_d = round(logP_dlipT*100)/100 ;
%calculate the membrane permeability

Pn = 10^(logP_n-6.7)*60; % in 1/min
Pd = 10^(logP_d-6.7)*60; % in 1/min

i = -sign(z) ;
%calculate N for flux of ion happening at 7 membranes
C = z*F/(R*T);
NaEp = C*(-EaEp) ;
NbEp = C*EbEp ;
NimEp = C*EimEp ;
Nsm = C*Esm ;
NimInt = C*EimInt ;
NbEd = C*(-EbEd) ;
NaEd = C*EaEd ;
%calculate Kn and Kd for 8 compartments
N = 1.22*10^(logP_n);
D = 1.22*10^(logP_d);
Kd =  D;

KaEpN = N*LaEp ;
KaEpD = D*LaEp ;
KimEpN = N*LimEp ;
KimEpD = Kd*LimEp ;
KcEpN = N*LcEp ;
KcEpD = Kd*LcEp ;
KintN = N*Lint ;
KintD = D*Lint ;
KsmN = N*Lsm ;
KsmD = Kd*Lsm ;
KimIntN = N*LimInt ;
KimIntD = Kd*LimInt ;
```

```
KcEdN = N*LcEd ;
KcEdD = Kd*LcEd ;
KpN = N*Lp ;
KpD = D*Lp ;


%####################for mito and lyso compartments in cEp, sm and
cEd####################################################
LcEpMito = 0.05 ;
LcEpLyso = 0.05 ;
LsmMito = 0.05 ;
LsmLyso = 0.05 ;
LcEdMito = 0.05 ;
LcEdLyso = 0.05 ;

WcEpMito = 1-LcEpMito ;
WcEpLyso = 1-LcEpLyso ;
WsmMito = 1-LsmMito ;
WsmLyso = 1-LsmLyso ;
WcEdMito = 1-LcEdMito ;
WcEdLyso = 1-LcEdLyso ;

GcEpMitoN = 1.23 ;
GcEpMitoD = 0.74 ;
GcEpLysoN = 1.23 ;
GcEpLysoD = 0.74 ;

GsmMitoN = 1.23 ;
GsmMitoD = 0.74 ;
GsmLysoN = 1.23 ;
GsmLysoD = 0.74 ;

GcEdMitoN = 1.23 ;
GcEdMitoD = 0.74 ;
GcEdLysoN = 1.23 ;
GcEdLysoD = 0.74 ;

EcEpMito = -0.16 ;
EcEpLyso = +0.01 ;
EsmMito = -0.16 ;
EsmLyso = +0.01 ;
EcEdMito = -0.16 ;
EcEdLyso = +0.01 ;

pHcEpMito = 8 ;
pHcEpLyso = 5 ;
pHsmMito = 8 ;
pHsmLyso = 5 ;
pHcEdMito = 8 ;
pHcEdLyso = 5 ;


NcEpMito = C*EcEpMito ;
NcEpLyso = C*EcEpLyso ;
NsmMito = C*EsmMito ;
NsmLyso = C*EsmLyso ;
```

```
NcEdMito = C*EcEdMito ;
NcEdLyso = C*EcEdLyso ;


KcEpMitoN = N*LcEpMito ;
KcEpMitoD = Kd*LcEpMito ;
KcEpLysoN = N*LcEpLyso ;
KcEpLysoD = Kd*LcEpLyso ;

KsmMitoN = N*LsmMito ;
KsmMitoD = Kd*LsmMito ;
KsmLysoN = N*LsmLyso ;
KsmLysoD = Kd*LsmLyso ;

KcEdMitoN = N*LcEdMito ;
KcEdMitoD = Kd*LcEdMito ;
KcEdLysoN = N*LcEdLyso ;
KcEdLysoD = Kd*LcEdLyso ;



fcEpMitoN = 1/(WcEpMito/GcEpMitoN+KcEpMitoN/GcEpMitoN+WcEpMito*10^(i*(pHcEpMito-
pKa))/GcEpMitoD...
        +KcEpMitoD*10^(i*(pHcEpMito-pKa))/GcEpMitoD);
fcEpMitoD = fcEpMitoN*10^(i*(pHcEpMito-pKa));

fcEpLysoN = 1/(WcEpLyso/GcEpLysoN+KcEpLysoN/GcEpLysoN+WcEpLyso*10^(i*(pHcEpLyso-
pKa))/GcEpLysoD...
        +KcEpLysoD*10^(i*(pHcEpLyso-pKa))/GcEpLysoD);
fcEpLysoD = fcEpLysoN*10^(i*(pHcEpLyso-pKa));


fsmMitoN = 1/(WsmMito/GsmMitoN+KsmMitoN/GsmMitoN+WsmMito*10^(i*(pHsmMito-pKa))/GsmMitoD...
        +KsmMitoD*10^(i*(pHsmMito-pKa))/GsmMitoD);
fsmMitoD = fsmMitoN*10^(i*(pHsmMito-pKa));

fsmLysoN = 1/(WsmLyso/GsmLysoN+KsmLysoN/GsmLysoN+WsmLyso*10^(i*(pHsmLyso-pKa))/GsmLysoD...
        +KsmLysoD*10^(i*(pHsmLyso-pKa))/GsmLysoD);
fsmLysoD = fsmLysoN*10^(i*(pHsmLyso-pKa));


fcEdMitoN = 1/(WcEdMito/GcEdMitoN+KcEdMitoN/GcEdMitoN+WcEdMito*10^(i*(pHcEdMito-
pKa))/GcEdMitoD...
        +KcEdMitoD*10^(i*(pHcEdMito-pKa))/GcEdMitoD);
fcEdMitoD = fcEdMitoN*10^(i*(pHcEdMito-pKa));

fcEdLysoN = 1/(WcEdLyso/GcEdLysoN+KcEdLysoN/GcEdLysoN+WcEdLyso*10^(i*(pHcEdLyso-
pKa))/GcEdLysoD...
        +KcEdLysoD*10^(i*(pHcEdLyso-pKa))/GcEdLysoD);
fcEdLysoD = fcEdLysoN*10^(i*(pHcEdLyso-pKa));

%###########################################################################

%compute the fn and fd for 8 compartments
faEpN = 1/(WaEp/GaEpN+KaEpN/GaEpN+WaEp*10^(i*(pHaEp-pKa))/GaEpD...
        +KaEpD*10^(i*(pHaEp-pKa))/GaEpD);
```

```
faEpD = faEpN*10^(i*(pHaEp-pKa));
fimEpN = 1/(WimEp/GimEpN+KimEpN/GimEpN+WimEp*10^(i*(pHimEp-pKa))/GimEpD...
        +KimEpD*10^(i*(pHimEp-pKa))/GimEpD);
fimEpD = fimEpN*10^(i*(pHimEp-pKa));
fcEpN = 1/(WcEp/GcEpN+KcEpN/GcEpN+WcEp*10^(i*(pHcEp-pKa))/GcEpD...
        +KcEpD*10^(i*(pHcEp-pKa))/GcEpD);
fcEpD = fcEpN*10^(i*(pHcEp-pKa));
fintN = 1/(Wint/GintN+KintN/GintN+Wint*10^(i*(pHint-pKa))/GintD...
        +KintD*10^(i*(pHint-pKa))/GintD);
fintD = fintN*10^(i*(pHint-pKa));
fimIntN = 1/(WimInt/GimIntN+KimIntN/GimIntN+WimInt*10^(i*(pHimInt-pKa))/GimIntD...
        +KimIntD*10^(i*(pHimInt-pKa))/GimIntD);
fimIntD = fimIntN*10^(i*(pHimInt-pKa));
fsmN = 1/(Wsm/GsmN+KsmN/GsmN+Wsm*10^(i*(pHsm-pKa))/GsmD...
        +KsmD*10^(i*(pHsm-pKa))/GsmD);
fsmD = fsmN*10^(i*(pHsm-pKa));
fcEdN = 1/(WcEd/GcEdN+KcEdN/GcEdN+WcEd*10^(i*(pHcEd-pKa))/GcEdD...
        +KcEdD*10^(i*(pHcEd-pKa))/GcEdD);
fcEdD = fcEdN*10^(i*(pHcEd-pKa));
fpN = 1/(Wp/GpN+KpN/GpN+Wp*10^(i*(pHp-pKa))/GpD...
        +KpD*10^(i*(pHp-pKa))/GpD);
fpD = fpN*10^(i*(pHp-pKa));

%mucus clearance
Ke = 0;

%compute the coefficient matrix for ODEs
% #1: Surface Lining Liquid (aEp)
KaEp_aEp = AaEp/VaEp*(Pn*(-faEpN)+Pd*NaEp/(exp(NaEp)-1)*(-faEpD)*exp(NaEp))...
      -AimEp/VaEp*(Pn*faEpN+Pd*NimEp/(exp(NimEp)-1)*faEpD)...
      -Ke;
KaEp_imEp = -AimEp/VaEp*(Pn*(-fimEpN)+Pd*NimEp/(exp(NimEp)-1)*(-fimEpD)*exp(NimEp));
KaEp_cEp = AaEp/VaEp*(Pn*(fcEpN)+Pd*NaEp/(exp(NaEp)-1)*(fcEpD));
KaEp_cEpMito = 0;
KaEp_cEpLyso = 0;
KaEp_int = 0;
KaEp_sm = 0;
KaEp_smMito = 0;
KaEp_smLyso = 0;
KaEp_imInt = 0;
KaEp_cEd = 0;
KaEp_cEdMito = 0;
KaEp_cEdLyso = 0;
KaEp_p = 0;
SaEp = 0;

% #2: Macrophage (imEp)
KimEp_aEp = AimEp/VimEp*(Pn*faEpN+Pd*NimEp/(exp(NimEp)-1)*faEpD);
KimEp_imEp = AimEp/VimEp*(Pn*(-fimEpN)+Pd*NimEp/(exp(NimEp)-1)*(-fimEpD)*exp(NimEp));
KimEp_cEp = 0;
KimEp_cEpMito = 0 ;
KimEp_cEpLyso = 0 ;
KimEp_int = 0;
KimEp_sm = 0;
KimEp_smMito = 0;
KimEp_smLyso = 0;
```

```
KimEp_imInt = 0;
KimEp_cEd = 0;
KimEp_cEdMito = 0 ;
KimEp_cEdLyso = 0 ;
KimEp_p = 0;
SimEp = 0;

% #3: Epithelial Cells (cEp)
KcEp_aEp = -AaEp/VcEp*(Pn*(-faEpN)+Pd*NaEp/(exp(NaEp)-1)*(-faEpD)*exp(NaEp));
KcEp_imEp = 0;
KcEp_cEp = -AaEp/VcEp*(Pn*(fcEpN)+Pd*NaEp/(exp(NaEp)-1)*(fcEpD))...
        -AcEpMito/VcEp*(Pn*fcEpN+Pd*NcEpMito/(exp(NcEpMito)-1)*fcEpD)...
        -AcEpLyso/VcEp*(Pn*fcEpN+Pd*NcEpLyso/(exp(NcEpLyso)-1)*fcEpD)...
        +AbEp/VcEp*(Pn*(-fcEpN)+Pd*NbEp/(exp(NbEp)-1)*(-fcEpD)*exp(NbEp));
KcEp_cEpMito = -AcEpMito/VcEp*(Pn*(-fcEpMitoN)+Pd*NcEpMito/(exp(NcEpMito)-1)*(-
fcEpMitoD)*exp(NcEpMito)) ;
KcEp_cEpLyso = -AcEpLyso/VcEp*(Pn*(-fcEpLysoN)+Pd*NcEpLyso/(exp(NcEpLyso)-1)*(-
fcEpLysoD)*exp(NcEpLyso)) ;
KcEp_int = AbEp/VcEp*(Pn*(fintN)+Pd*NbEp/(exp(NbEp)-1)*(fintD));
KcEp_sm = 0;
KcEP_smMito = 0;
KcEp_smLyso = 0;
KcEp_imInt = 0;
KcEp_cEd = 0;
KcEp_cEdMito = 0 ;
KcEp_cEdLyso = 0 ;
KcEp_p = 0;
ScEp = 0;

% #4: : Epithelial Cells (cEpMito)
KcEpMito_aEp = 0;
KcEpMito_imEp = 0;
KcEpMito_cEp = AcEpMito/VcEpMito*(Pn*(fcEpN)+Pd*NcEpMito/(exp(NcEpMito)-1)*(fcEpD));
KcEpMito_cEpMito = AcEpMito/VcEpMito*(Pn*(-fcEpMitoN)+Pd*NcEpMito/(exp(NcEpMito)-1)*(-
fcEpMitoD)*exp(NcEpMito));
KcEpMito_cEpLyso = 0 ;
KcEpMito_int = 0 ;
KcEpMito_sm = 0;
KcEpMito_smMito = 0;
KcEpMito_smLyso = 0;
KcEpMito_imInt = 0;
KcEpMito_cEd = 0;
KcEpMito_cEdMito = 0 ;
KcEpMito_cEdLyso = 0 ;
KcEpMito_p = 0;
ScEpMito = 0;

% #5: : Epithelial Cells (cEpLyso)
KcEpLyso_aEp = 0;
KcEpLyso_imEp = 0;
KcEpLyso_cEp = AcEpLyso/VcEpLyso*(Pn*(fcEpN)+Pd*NcEpLyso/(exp(NcEpLyso)-1)*(fcEpD));
KcEpLyso_cEpMito = 0 ;
KcEpLyso_cEpLyso = AcEpLyso/VcEpLyso*(Pn*(-fcEpLysoN)+Pd*NcEpLyso/(exp(NcEpLyso)-1)*(-
fcEpLysoD)*exp(NcEpLyso));
KcEpLyso_int = 0 ;
KcEpLyso_sm = 0;
```

```
KcEpLyso_smMito = 0;
KcEpLyso_smLyso = 0;
KcEpLyso_imInt = 0;
KcEpLyso_cEd = 0;
KcEpLyso_cEdMito = 0 ;
KcEpLyso_cEdLyso = 0 ;
KcEpLyso_p = 0;
ScEpLyso = 0;

% #6: : Interstitium (int)
Kint_aEp = 0;
Kint_imEp = 0;
Kint_cEp = -AbEp/Vint*(Pn*(-fcEpN)+Pd*NbEp/(exp(NbEp)-1)*(-fcEpD)*exp(NbEp));
Kint_cEpMito = 0 ;
Kint_cEpLyso = 0 ;
Kint_int = -AbEp/Vint*(Pn*(fintN)+Pd*NbEp/(exp(NbEp)-1)*(fintD))...
        -Asm/Vint*(Pn*fintN+Pd*Nsm/(exp(Nsm)-1)*fintD)...
        -AimInt/Vint*(Pn*fintN+Pd*NimInt/(exp(NimInt)-1)*fintD)...
        +AbEd/Vint*(Pn*(-fintN)+Pd*NbEd/(exp(NbEd)-1)*(-fintD)*exp(NbEd));
Kint_sm = -Asm/Vint*(Pn*(-fsmN)+Pd*Nsm/(exp(Nsm)-1)*(-fsmD)*exp(Nsm));
Kint_smMito = 0;
Kint_smLyso = 0;
Kint_imInt = -AimInt/Vint*(Pn*(-fimIntN)+Pd*NimInt/(exp(NimInt)-1)*(-fimIntD)*exp(NimInt));
Kint_cEd = AbEd/Vint*(Pn*(fcEdN)+Pd*NbEd/(exp(NbEd)-1)*(fcEdD));
Kint_cEdMito = 0 ;
Kint_cEdLyso = 0 ;
Kint_p = 0;
Sint = 0;


% #7: Smooth Muscle (sm)
Ksm_aEp = 0;
Ksm_imEp = 0;
Ksm_cEp = 0;
Ksm_cEpMito = 0 ;
Ksm_cEpLyso = 0 ;
Ksm_int = Asm/Vsm*(Pn*fintN+Pd*Nsm/(exp(Nsm)-1)*fintD);
Ksm_sm = Asm/Vsm*(Pn*(-fsmN)+Pd*Nsm/(exp(Nsm)-1)*(-fsmD)*exp(Nsm))...
       -AsmMito/Vsm*(Pn*fsmN+Pd*NsmMito/(exp(NsmMito)-1)*fsmD)...
       -AsmLyso/Vsm*(Pn*fsmN+Pd*NsmLyso/(exp(NsmLyso)-1)*fsmD);
Ksm_smMito = -AsmMito/Vsm*(Pn*(-fsmMitoN)+Pd*NsmMito/(exp(NsmMito)-1)*(-
fsmMitoD)*exp(NsmMito)) ;
Ksm_smLyso = -AsmLyso/Vsm*(Pn*(-fsmLysoN)+Pd*NsmLyso/(exp(NsmLyso)-1)*(-
fsmLysoD)*exp(NsmLyso)) ;
Ksm_imInt = 0;
Ksm_cEd = 0;
Ksm_cEdMito = 0 ;
Ksm_cEdLyso = 0 ;
Ksm_p = 0;
Ssm = 0;

% #8: Smooth Muscle (smMito)
KsmMito_aEp = 0;
KsmMito_imEp = 0;
KsmMito_cEp = 0;
KsmMito_cEpMito = 0;
```

```
KsmMito_cEpLyso = 0 ;
KsmMito_int = 0 ;
KsmMito_sm = AsmMito/VsmMito*(Pn*(fsmN)+Pd*NsmMito/(exp(NsmMito)-1)*(fsmD));
KsmMito_smMito = AsmMito/VsmMito*(Pn*(-fsmMitoN)+Pd*NsmMito/(exp(NsmMito)-1)*(-
fsmMitoD)*exp(NsmMito));
KsmMito_smLyso = 0;
KsmMito_imInt = 0;
KsmMito_cEd = 0;
KsmMito_cEdMito = 0 ;
KsmMito_cEdLyso = 0 ;
KsmMito_p = 0;
SsmMito = 0;

% #9: Smooth Muscle (smLyso)
KsmLyso_aEp = 0;
KsmLyso_imEp = 0;
KsmLyso_cEp = 0;
KsmLyso_cEpMito = 0 ;
KsmLyso_cEpLyso = 0;
KsmLyso_int = 0 ;
KsmLyso_sm = AsmLyso/VsmLyso*(Pn*(fsmN)+Pd*NsmLyso/(exp(NsmLyso)-1)*(fsmD));
KsmLyso_smMito = 0;
KsmLyso_smLyso = AsmLyso/VsmLyso*(Pn*(-fsmLysoN)+Pd*NsmLyso/(exp(NsmLyso)-1)*(-
fsmLysoD)*exp(NsmLyso));
KsmLyso_imInt = 0;
KsmLyso_cEd = 0;
KsmLyso_cEdMito = 0 ;
KsmLyso_cEdLyso = 0 ;
KsmLyso_p = 0;
SsmLyso = 0;

% #10: Immune Cells (imInt)
KimInt_aEp = 0;
KimInt_imEp = 0;
KimInt_cEp = 0;
KimInt_cEpMito = 0;
KimInt_cEpLyso = 0;
KimInt_int = AimInt/VimInt*(Pn*fintN+Pd*NimInt/(exp(NimInt)-1)*fintD);
KimInt_sm = 0;
KimInt_smMito = 0;
KimInt_smLyso = 0;
KimInt_imInt = AimInt/VimInt*(Pn*(-fimIntN)+Pd*NimInt/(exp(NimInt)-1)*(-fimIntD)*exp(NimInt));
KimInt_cEd = 0;
KimInt_cEdMito = 0;
KimInt_cEdLyso = 0;
KimInt_p = 0;
SimInt = 0;

% #11: Endothelial celss (cEd)
KcEd_aEp = 0;
KcEd_imEp = 0;
KcEd_cEp = 0;
KcEd_cEpMito = 0;
KcEd_cEpLyso = 0;
KcEd_int = -AbEd/VcEd*(Pn*(-fintN)+Pd*NbEd/(exp(NbEd)-1)*(-fintD)*exp(NbEd));
KcEd_sm = 0;
```

```
KcEd_smMito = 0;
KcEd_smLyso = 0;
KcEd_imInt = 0;
KcEd_cEd = -AbEd/VcEd*(Pn*(fcEdN)+Pd*NbEd/(exp(NbEd)-1)*(fcEdD))...
        -AcEdMito/VcEd*(Pn*fcEdN+Pd*NcEdMito/(exp(NcEdMito)-1)*fcEdD)...
        -AcEdLyso/VcEd*(Pn*fcEdN+Pd*NcEdLyso/(exp(NcEdLyso)-1)*fcEdD)...
        +AaEd/VcEd*(Pn*(-fcEdN)+Pd*NaEd/(exp(NaEd)-1)*(-fcEdD)*exp(NaEd));
KcEd_cEdMito = -AcEdMito/VcEd*(Pn*(-fcEdMitoN)+Pd*NcEdMito/(exp(NcEdMito)-1)*(-
fcEdMitoD)*exp(NcEdMito));
KcEd_cEdLyso = -AcEdLyso/VcEd*(Pn*(-fcEdLysoN)+Pd*NcEdLyso/(exp(NcEdLyso)-1)*(-
fcEdLysoD)*exp(NcEdLyso));
KcEd_p = AaEd/VcEd*(Pn*(fpN)+Pd*NaEd/(exp(NaEd)-1)*(fpD));
ScEd = 0;

% #12: Endothelial celss (cEd) Mito
KcEdMito_aEp = 0;
KcEdMito_imEp = 0;
KcEdMito_cEp = 0;
KcEdMito_cEpMito = 0;
KcEdMito_cEpLyso = 0;
KcEdMito_int = 0;
KcEdMito_sm = 0;
KcEdMito_smMito = 0;
KcEdMito_smLyso = 0;
KcEdMito_imInt = 0;
KcEdMito_cEd = AcEdMito/VcEdMito*(Pn*(fcEdN)+Pd*NcEdMito/(exp(NcEdMito)-1)*(fcEdD)) ;
KcEdMito_cEdMito = AcEdMito/VcEdMito*(Pn*(-fcEdMitoN)+Pd*NcEdMito/(exp(NcEdMito)-1)*(-
fcEdMitoD)*exp(NcEdMito));
KcEdMito_cEdLyso = 0;
KcEdMito_p = 0 ;
ScEdMito = 0;

% #13: Endothelial celss (cEd) Lyso
KcEdLyso_aEp = 0;
KcEdLyso_imEp = 0;
KcEdLyso_cEp = 0;
KcEdLyso_cEpMito = 0;
KcEdLyso_cEpLyso = 0;
KcEdLyso_int = 0 ;
KcEdLyso_sm = 0;
KcEdLyso_smMito = 0;
KcEdLyso_smLyso = 0;
KcEdLyso_imInt = 0;
KcEdLyso_cEd = AcEdLyso/VcEdLyso*(Pn*(fcEdN)+Pd*NcEdLyso/(exp(NcEdLyso)-1)*(fcEdD)) ;
KcEdLyso_cEdMito = 0;
KcEdLyso_cEdLyso = AcEdLyso/VcEdLyso*(Pn*(-fcEdLysoN)+Pd*NcEdLyso/(exp(NcEdLyso)-1)*(-
fcEdLysoD)*exp(NcEdLyso));
KcEdLyso_p = 0;
ScEdLyso = 0;

% #14: plasma(p)
Kp_aEp = 0;
Kp_imEp = 0;
Kp_cEp = 0;
Kp_cEpMito = 0;
Kp_cEpLyso = 0;
```

```
Kp_int = 0;
Kp_sm = 0;
Kp_smMito = 0;
Kp_smLyso = 0;
Kp_imInt = 0;
Kp_cEd = -AaEd/Vp*(Pn*(-fcEdN)+Pd*NaEd/(exp(NaEd)-1)*(-fcEdD)*exp(NaEd));
Kp_cEdMito = 0;
Kp_cEdLyso = 0;
Kp_p = -AaEd/Vp*(Pn*(fpN)+Pd*NaEd/(exp(NaEd)-1)*(fpD));
Sp = 0;

M =
[KaEp_aEp,KaEp_imEp,KaEp_cEp,KaEp_cEpMito,KaEp_cEpLyso,KaEp_int,KaEp_sm,KaEp_smMito,KaEp_smL
yso,KaEp_imInt,KaEp_cEd,KaEp_cEdMito,KaEp_cEdLyso,KaEp_p;...

KimEp_aEp,KimEp_imEp,KimEp_cEp,KimEp_cEpMito,KimEp_cEpLyso,KimEp_int,KimEp_sm,KimEp_smMito,
KimEp_smLyso,KimEp_imInt,KimEp_cEd,KimEp_cEdMito,KimEp_cEdLyso,KimEp_p;...

KcEp_aEp,KcEp_imEp,KcEp_cEp,KcEp_cEpMito,KcEp_cEpLyso,KcEp_int,KcEp_sm,KcEP_smMito,KcEp_smL
yso,KcEp_imInt,KcEp_cEd,KcEp_cEdMito,KcEp_cEdLyso,KcEp_p;...

KcEpMito_aEp,KcEpMito_imEp,KcEpMito_cEp,KcEpMito_cEpMito,KcEpMito_cEpLyso,KcEpMito_int,KcEpMi
to_sm,KcEpMito_smMito,KcEpMito_smLyso,KcEpMito_imInt,KcEpMito_cEd,KcEpMito_cEdMito,KcEpMito_c
EdLyso,KcEpMito_p;...

KcEpLyso_aEp,KcEpLyso_imEp,KcEpLyso_cEp,KcEpLyso_cEpMito,KcEpLyso_cEpLyso,KcEpLyso_int,KcEpL
yso_sm,KcEpLyso_smMito,KcEpLyso_smLyso,KcEpLyso_imInt,KcEpLyso_cEd,KcEpLyso_cEdMito,KcEpLyso_
cEdLyso,KcEpLyso_p;...

Kint_aEp,Kint_imEp,Kint_cEp,Kint_cEpMito,Kint_cEpLyso,Kint_int,Kint_sm,Kint_smMito,Kint_smLyso,Kint_i
mInt,Kint_cEd,Kint_cEdMito,Kint_cEdLyso,Kint_p;...

Ksm_aEp,Ksm_imEp,Ksm_cEp,Ksm_cEpMito,Ksm_cEpLyso,Ksm_int,Ksm_sm,Ksm_smMito,Ksm_smLyso,Ksm
_imInt,Ksm_cEd,Ksm_cEdMito,Ksm_cEdLyso,Ksm_p;...

KsmMito_aEp,KsmMito_imEp,KsmMito_cEp,KsmMito_cEpMito,KsmMito_cEpLyso,KsmMito_int,KsmMito_sm,
KsmMito_smMito,KsmMito_smLyso,KsmMito_imInt,KsmMito_cEd,KsmMito_cEdMito,KsmMito_cEdLyso,Ksm
Mito_p;...

KsmLyso_aEp,KsmLyso_imEp,KsmLyso_cEp,KsmLyso_cEpMito,KsmLyso_cEpLyso,KsmLyso_int,KsmLyso_sm
,KsmLyso_smMito,KsmLyso_smLyso,KsmLyso_imInt,KsmLyso_cEd,KsmLyso_cEdMito,KsmLyso_cEdLyso,Ks
mLyso_p;...

KimInt_aEp,KimInt_imEp,KimInt_cEp,KimInt_cEpMito,KimInt_cEpLyso,KimInt_int,KimInt_sm,KimInt_smMito,
KimInt_smLyso,KimInt_imInt,KimInt_cEd,KimInt_cEdMito,KimInt_cEdLyso,KimInt_p;...

KcEd_aEp,KcEd_imEp,KcEd_cEp,KcEd_cEpMito,KcEd_cEpLyso,KcEd_int,KcEd_sm,KcEd_smMito,KcEd_smL
yso,KcEd_imInt,KcEd_cEd,KcEd_cEdMito,KcEd_cEdLyso,KcEd_p;...

KcEdMito_aEp,KcEdMito_imEp,KcEdMito_cEp,KcEdMito_cEpMito,KcEdMito_cEpLyso,KcEdMito_int,KcEdMi
to_sm,KcEdMito_smMito,KcEdMito_smLyso,KcEdMito_imInt,KcEdMito_cEd,KcEdMito_cEdMito,KcEdMito_c
EdLyso,KcEdMito_p;...

KcEdLyso_aEp,KcEdLyso_imEp,KcEdLyso_cEp,KcEdLyso_cEpMito,KcEdLyso_cEpLyso,KcEdLyso_int,KcEdL
yso_sm,KcEdLyso_smMito,KcEdLyso_smLyso,KcEdLyso_imInt,KcEdLyso_cEd,KcEdLyso_cEdMito,KcEdLyso_
cEdLyso,KcEdLyso_p;...
```

Kp_aEp,Kp_imEp,Kp_cEp,Kp_cEpMito,Kp_cEpLyso,Kp_int,Kp_sm,Kp_smMito,Kp_smLyso,Kp_imInt,Kp_cEd,
Kp_cEdMito,Kp_cEdLyso,Kp_p];

G = [SaEp,SimEp,ScEp,ScEpMito,ScEpLyso,Sint,Ssm,SsmMito,SsmLyso,SimInt,ScEd,ScEdMito,ScEdLyso,Sp]';

```
% 4/30/2010 J YU
% 1CellPK based lung:Alveoli model starts here (Rats)
% 12 compartments:
% aEp (surface lining liquied), imEp (Macrophage),
% cEp(epithelial cells),cEpMito(mito of cEp), cEpLyso (lyso of cEp)
% int(Interstitium),imInt(immune cells), sm(smooth muscle),
% cEd(endothelial cells), cEdMito(mito of cEd),cEdLyso (lyso of cEd), p(plasma)

function [M, G, M_v, Vp] = H_al_RL()

%molecular physiochemical property

pKa = 7.87;
logPN = 4 ;
logPD = logPN-3.7 ;
z = 1;
% %weighted

% Constant
T = 273.15+37;
R = 8.314;
F = 96484.56;
%lipid fraction
LaEp = 0.95;
LimEp = 0.05;
LcEp = 0.05 ;
Lint = 0;
LimInt = 0.05;
Lsm = 0;
LcEd = 0.05;
Lp = 0;
%volumetric water fraction=1-lipid fraction
WaEp = 1 - LaEp;
WimEp = 1 - LimEp;
WcEp = 1 - LcEp;
Wint = 1 - Lint;
WimInt = 1 - LimInt;
Wsm = 1 - Lsm;
WcEd = 1 - LcEd;
Wp = 1 - Lp;
%activity coefficient of species(N:neutral,D:desociated)
GaEpN = 1;
GaEpD = 1;
GimEpN = 1.23;
GimEpD = 0.74;
GcEpN = 1.23;
GcEpD = 0.74;
GintN = 1;
GintD = 1;
GimIntN = 1.23;
GimIntD = 0.74;
GsmN = 1.23;
GsmD = 0.74;
GcEdN = 1.23;
GcEdD = 0.74;
GpN = 1;
```

```
GpD = 1;

% By Jingyu Yu (used in publication) Areas and volumes (m^2, m^3) for 7 membranes and corresponding
compartments
AaEp = 0.387;%literature
AbEp = AaEp;%Assuming same with epical side
AimEp = 3.14*10^(-10)*0.89*10^(9)*3/100/2; % 10 um diameter, only half of surface gets contact with liquid,
since ASL = 5 um
AimInt = AimEp/10; % assuming number of immune cells is 1/10 of macrophage
Asm = 0;%No SM
AbEd = 0.452;%literature
AaEd = 0.452;%literature
%volumes for 8 compartments(m3)
ASL = 5; %literature um
VaEp = AaEp*ASL*10^(-6); %5 um thickness
VcEp = AaEp*0.384*10^(-6); % 0.384, literature
VimEp = 0.89*10^(9)*3/100*1058*10^(-18);%number of macrophage(literature)*volume of macrophage
Vint = AaEp*0.693*10^(-6); % literature
VimInt = VimEp/10;% assuming number of immune cells is 1/10 of macrophage
Vsm = 10^(-30); % VcEp*10^(-12); % can be any number, surface is 0
VcEd = AbEd*0.358*10^(-6); %0.358 um thickness --literature
% Vp = 5; %total huge volume for lung absorption model

%########################################################################
% Subcellular compartments in cEp (epithelial cells) and cEd(endothelial cells)
% calculate constant
R_org = 0.1;

VcEpMito = R_org*VcEp ; % 10^(-30); %
VcEpLyso = R_org*VcEp ; % 10^(-30); %
VcEdMito = R_org*VcEd ; % 10^(-30); %
VcEdLyso = R_org*VcEd ; % 10^(-30); %

AcEpMito = 5.9924e+006*VcEpMito; % 0 ;
AcEpLyso = 5.9924e+006*VcEpLyso; % 0 ;
AcEdMito = 5.9924e+006*VcEdMito; % 0 ;
AcEdLyso = 5.9924e+006*VcEdLyso; % 0 ;
%########################################################################

M_v = diag([VaEp,VimEp,VcEp,VcEpMito,VcEpLyso,Vint,Vsm,VimInt,VcEd,VcEdMito,VcEdLyso]);
V_LUN = trace(M_v)*10^6;
Vp = 340*10^(-9)*V_LUN;

% Membrane potential (V)
EaEp = -0.0093;
EbEp = 0.0119;%0.0119;
EimEp = -0.06;
EimInt = -0.06;
Esm = -0.06;
EbEd = -0.06;
EaEd = -0.06;
% pH values
pHaEp = 7.4;
pHimEp = 7.0;
pHcEp = 7.0;
pHint = 7.0;
```

```
pHimInt = 7.0;
pHsm = 7.0;
pHcEd = 7.0;
pHp = 7.4;

%adjustment for logP
if ( abs(z-1) <= 10^(-6) )
    logP_nlipT = 0.33*logPN+2.2 ;
    logP_dlipT = 0.37*logPD+2 ;
end
if ( abs(z+1) <= 10^(-6) )
    logP_nlipT = 0.37*logPN+2.2 ;
    logP_dlipT = 0.33*logPD+2.6 ;
end
if ( abs(z-0) <= 10^(-5) )
    logP_nlipT = 0.33*logPN+2.2 ;
    logP_dlipT = 0.33*logPD+2.2 ;
end

% Get the first two decimals
logP_n = round(logP_nlipT*100)/100 ;
logP_d = round(logP_dlipT*100)/100 ;
%calculate the membrane permeability

Pn = 10^(logP_n-6.7)*60; % in 1/min
Pd = 10^(logP_d-6.7)*60; % in 1/min

i = -sign(z) ;
%calculate N for flux of ion happening at 7 membranes
C = z*F/(R*T);
NaEp = C*(-EaEp) ;
NbEp = C*EbEp ;
NimEp = C*EimEp ;
NimInt = C*EimInt ;
Nsm = C*Esm ;
NbEd = C*(-EbEd) ;
NaEd = C*(EaEd) ;

%calculate Kn and Kd for 8 compartments
N = 1.22*10^(logP_n);
D = 1.22*10^(logP_d);
Kd =  D;

KaEpN = N*LaEp ;
KaEpD = D*LaEp ;
KimEpN = N*LimEp ;
KimEpD = Kd*LimEp;
KcEpN = N*LcEp ;
KcEpD = Kd*LcEp ;
KintN = N*Lint ;
KintD = D*Lint ;
KimIntN = N*LimInt ;
KimIntD = Kd*LimInt ;
KsmN = N*Lsm ;
KsmD = Kd*Lsm ;
KcEdN = N*LcEd ;
```

```
KcEdD = Kd*LcEd ;
KpN = N*Lp ;
KpD = D*Lp ;


%##########################################################################
LcEpMito = 0.05 ;
LcEpLyso = 0.05 ;
LcEdMito = 0.05 ;
LcEdLyso = 0.05 ;

WcEpMito = 1-LcEpMito ;
WcEpLyso = 1-LcEpLyso ;
WcEdMito = 1-LcEdMito ;
WcEdLyso = 1-LcEdLyso ;

GcEpMitoN = 1.23 ;
GcEpMitoD = 0.74 ;
GcEpLysoN = 1.23 ;
GcEpLysoD = 0.74 ;
GcEdMitoN = 1.23 ;
GcEdMitoD = 0.74 ;
GcEdLysoN = 1.23 ;
GcEdLysoD = 0.74 ;

EcEpMito = -0.16 ;
EcEpLyso = +0.01 ;
EcEdMito = -0.16 ;
EcEdLyso = +0.01 ;

pHcEpMito = 8 ;
pHcEpLyso = 5 ;
pHcEdMito = 8 ;
pHcEdLyso = 5 ;


NcEpMito = C*EcEpMito ;
NcEpLyso = C*EcEpLyso ;
NcEdMito = C*EcEdMito ;
NcEdLyso = C*EcEdLyso ;



KcEpMitoN = N*LcEpMito ;
KcEpMitoD = Kd*LcEpMito ;
KcEpLysoN = N*LcEpLyso ;
KcEpLysoD = Kd*LcEpLyso ;

KcEdMitoN = N*LcEdMito ;
KcEdMitoD = Kd*LcEdMito ;
KcEdLysoN = N*LcEdLyso ;
KcEdLysoD = Kd*LcEdLyso ;

fcEpMitoN = 1/(WcEpMito/GcEpMitoN+KcEpMitoN/GcEpMitoN+WcEpMito*10^(i*(pHcEpMito-
pKa))/GcEpMitoD...
      +KcEpMitoD*10^(i*(pHcEpMito-pKa))/GcEpMitoD);
```

```
fcEpMitoD = fcEpMitoN*10^(i*(pHcEpMito-pKa));

fcEpLysoN = 1/(WcEpLyso/GcEpLysoN+KcEpLysoN/GcEpLysoN+WcEpLyso*10^(i*(pHcEpLyso-
pKa))/GcEpLysoD...
        +KcEpLysoD*10^(i*(pHcEpLyso-pKa))/GcEpLysoD);
fcEpLysoD = fcEpLysoN*10^(i*(pHcEpLyso-pKa));

fcEdMitoN = 1/(WcEdMito/GcEdMitoN+KcEdMitoN/GcEdMitoN+WcEdMito*10^(i*(pHcEdMito-
pKa))/GcEdMitoD...
        +KcEdMitoD*10^(i*(pHcEdMito-pKa))/GcEdMitoD);
fcEdMitoD = fcEdMitoN*10^(i*(pHcEdMito-pKa));

fcEdLysoN = 1/(WcEdLyso/GcEdLysoN+KcEdLysoN/GcEdLysoN+WcEdLyso*10^(i*(pHcEdLyso-
pKa))/GcEdLysoD...
        +KcEdLysoD*10^(i*(pHcEdLyso-pKa))/GcEdLysoD);
fcEdLysoD = fcEdLysoN*10^(i*(pHcEdLyso-pKa));

%############################################################################

%compute the fn and fd for 8 compartments
faEpN = 1/(WaEp/GaEpN+KaEpN/GaEpN+WaEp*10^(i*(pHaEp-pKa))/GaEpD...
        +KaEpD*10^(i*(pHaEp-pKa))/GaEpD);
faEpD = faEpN*10^(i*(pHaEp-pKa));
fimEpN = 1/(WimEp/GimEpN+KimEpN/GimEpN+WimEp*10^(i*(pHimEp-pKa))/GimEpD...
        +KimEpD*10^(i*(pHimEp-pKa))/GimEpD);
fimEpD = fimEpN*10^(i*(pHimEp-pKa));
fcEpN = 1/(WcEp/GcEpN+KcEpN/GcEpN+WcEp*10^(i*(pHcEp-pKa))/GcEpD...
        +KcEpD*10^(i*(pHcEp-pKa))/GcEpD);
fcEpD = fcEpN*10^(i*(pHcEp-pKa));
fintN = 1/(Wint/GintN+KintN/GintN+Wint*10^(i*(pHint-pKa))/GintD...
        +KintD*10^(i*(pHint-pKa))/GintD);
fintD = fintN*10^(i*(pHint-pKa));
fimIntN = 1/(WimInt/GimIntN+KimIntN/GimIntN+WimInt*10^(i*(pHimInt-pKa))/GimIntD...
        +KimIntD*10^(i*(pHimInt-pKa))/GimIntD);
fimIntD = fimIntN*10^(i*(pHimInt-pKa));
fsmN = 1/(Wsm/GsmN+KsmN/GsmN+Wsm*10^(i*(pHsm-pKa))/GsmD...
        +KsmD*10^(i*(pHsm-pKa))/GsmD);
fsmD = fsmN*10^(i*(pHsm-pKa));
fcEdN = 1/(WcEd/GcEdN+KcEdN/GcEdN+WcEd*10^(i*(pHcEd-pKa))/GcEdD...
        +KcEdD*10^(i*(pHcEd-pKa))/GcEdD);
fcEdD = fcEdN*10^(i*(pHcEd-pKa));
fpN = 1/(Wp/GpN+KpN/GpN+Wp*10^(i*(pHp-pKa))/GpD...
        +KpD*10^(i*(pHp-pKa))/GpD);
fpD = fpN*10^(i*(pHp-pKa));

%mucus clearance
%Ke = 0.02;
Ke = 0;

%compute the coefficient matrix for ODEs
% #1: Surface Lining Liquid (aEp)
KaEp_aEp = AaEp/VaEp*(Pn*(-faEpN)+Pd*NaEp/(exp(NaEp)-1)*(-faEpD)*exp(NaEp))...
       -AimEp/VaEp*(Pn*faEpN+Pd*NimEp/(exp(NimEp)-1)*faEpD)...
       -Ke;
KaEp_imEp = -AimEp/VaEp*(Pn*(-fimEpN)+Pd*NimEp/(exp(NimEp)-1)*(-fimEpD)*exp(NimEp));
KaEp_cEp = AaEp/VaEp*(Pn*(fcEpN)+Pd*NaEp/(exp(NaEp)-1)*(fcEpD));
```

```
KaEp_cEpMito = 0;
KaEp_cEpLyso = 0;
KaEp_int = 0;
KaEp_sm = 0;
KaEp_imInt = 0;
KaEp_cEd = 0;
KaEp_cEdMito = 0;
KaEp_cEdLyso = 0;
KaEp_p = 0;
SaEp = 0;

% #2: Macrophage (imEp)
KimEp_aEp = AimEp/VimEp*(Pn*faEpN+Pd*NimEp/(exp(NimEp)-1)*faEpD);
KimEp_imEp = AimEp/VimEp*(Pn*(-fimEpN)+Pd*NimEp/(exp(NimEp)-1)*(-fimEpD)*exp(NimEp));
KimEp_cEp = 0;
KimEp_cEpMito = 0 ;
KimEp_cEpLyso = 0 ;
KimEp_int = 0;
KimEp_sm = 0;
KimEp_imInt = 0;
KimEp_cEd = 0;
KimEp_cEdMito = 0 ;
KimEp_cEdLyso = 0 ;
KimEp_p = 0;
SimEp = 0;

% #3: Epithelial Cells (cEp)
KcEp_aEp = -AaEp/VcEp*(Pn*(-faEpN)+Pd*NaEp/(exp(NaEp)-1)*(-faEpD)*exp(NaEp));
KcEp_imEp = 0;
KcEp_cEp = -AaEp/VcEp*(Pn*(fcEpN)+Pd*NaEp/(exp(NaEp)-1)*(fcEpD))...
        -AcEpMito/VcEp*(Pn*fcEpN+Pd*NcEpMito/(exp(NcEpMito)-1)*fcEpD)...
        -AcEpLyso/VcEp*(Pn*fcEpN+Pd*NcEpLyso/(exp(NcEpLyso)-1)*fcEpD)...
        + AbEp/VcEp*(Pn*(-fcEpN)+Pd*NbEp/(exp(NbEp)-1)*(-fcEpD)*exp(NbEp));
KcEp_cEpMito = -AcEpMito/VcEp*(Pn*(-fcEpMitoN)+Pd*NcEpMito/(exp(NcEpMito)-1)*(-
fcEpMitoD)*exp(NcEpMito)) ;
KcEp_cEpLyso = -AcEpLyso/VcEp*(Pn*(-fcEpLysoN)+Pd*NcEpLyso/(exp(NcEpLyso)-1)*(-
fcEpLysoD)*exp(NcEpLyso)) ;
KcEp_int = AbEp/VcEp*(Pn*(fintN)+Pd*NbEp/(exp(NbEp)-1)*(fintD));
KcEp_sm = 0;
KcEp_imInt = 0;
KcEp_cEd = 0;
KcEp_cEdMito = 0 ;
KcEp_cEdLyso = 0 ;
KcEp_p = 0;
ScEp = 0;

% #4: : Epithelial Cells (cEpMito)
KcEpMito_aEp = 0;
KcEpMito_imEp = 0;
KcEpMito_cEp = AcEpMito/VcEpMito*(Pn*(fcEpN)+Pd*NcEpMito/(exp(NcEpMito)-1)*(fcEpD));
KcEpMito_cEpMito = AcEpMito/VcEpMito*(Pn*(-fcEpMitoN)+Pd*NcEpMito/(exp(NcEpMito)-1)*(-
fcEpMitoD)*exp(NcEpMito));
KcEpMito_cEpLyso = 0 ;
KcEpMito_int = 0 ;
KcEpMito_sm = 0;
KcEpMito_imInt = 0;
```

```
KcEpMito_cEd = 0;
KcEpMito_cEdMito = 0 ;
KcEpMito_cEdLyso = 0 ;
KcEpMito_p = 0;
ScEpMito = 0;

% #5: : Epithelial Cells (cEpLyso)
KcEpLyso_aEp = 0;
KcEpLyso_imEp = 0;
KcEpLyso_cEp = AcEpLyso/VcEpLyso*(Pn*(fcEpN)+Pd*NcEpLyso/(exp(NcEpLyso)-1)*(fcEpD));
KcEpLyso_cEpMito = 0 ;
KcEpLyso_cEpLyso = AcEpLyso/VcEpLyso*(Pn*(-fcEpLysoN)+Pd*NcEpLyso/(exp(NcEpLyso)-1)*(-
fcEpLysoD)*exp(NcEpLyso));
KcEpLyso_int = 0 ;
KcEpLyso_sm = 0;
KcEpLyso_imInt = 0;
KcEpLyso_cEd = 0;
KcEpLyso_cEdMito = 0 ;
KcEpLyso_cEdLyso = 0 ;
KcEpLyso_p = 0;
ScEpLyso = 0;


% #6: : Interstitium (int)
Kint_aEp = 0;
Kint_imEp = 0;
Kint_cEp = -AbEp/Vint*(Pn*(-fcEpN)+Pd*NbEp/(exp(NbEp)-1)*(-fcEpD)*exp(NbEp));
Kint_cEpMito = 0 ;
Kint_cEpLyso = 0 ;
Kint_int = -AbEp/Vint*(Pn*(fintN)+Pd*NbEp/(exp(NbEp)-1)*(fintD))...
    -Asm/Vint*(Pn*fintN+Pd*Nsm/(exp(Nsm)-1)*fintD)...
    -AimInt/Vint*(Pn*fintN+Pd*NimInt/(exp(NimInt)-1)*fintD)...
    +AbEd/Vint*(Pn*(-fintN)+Pd*NbEd/(exp(NbEd)-1)*(-fintD)*exp(NbEd));
Kint_sm = -Asm/Vint*(Pn*(-fsmN)+Pd*Nsm/(exp(Nsm)-1)*(-fsmD)*exp(Nsm));
Kint_imInt = -AimInt/Vint*(Pn*(-fimIntN)+Pd*NimInt/(exp(NimInt)-1)*(-fimIntD)*exp(NimInt));
Kint_cEd = AbEd/Vint*(Pn*(fcEdN)+Pd*NbEd/(exp(NbEd)-1)*(fcEdD));
Kint_cEdMito = 0 ;
Kint_cEdLyso = 0 ;
Kint_p = 0;
Sint = 0;

% #7: Smooth Muscle (sm)
Ksm_aEp = 0;
Ksm_imEp = 0;
Ksm_cEp = 0;
Ksm_cEpMito = 0 ;
Ksm_cEpLyso = 0 ;
Ksm_int = Asm/Vsm*(Pn*fintN+Pd*Nsm/(exp(Nsm)-1)*fintD);
Ksm_sm = Asm/Vsm*(Pn*(-fsmN)+Pd*Nsm/(exp(Nsm)-1)*(-fsmD)*exp(Nsm));
Ksm_imInt = 0;
Ksm_cEd = 0;
Ksm_cEdMito = 0 ;
Ksm_cEdLyso = 0 ;
Ksm_p = 0;
Ssm = 0;
```

```
% #8: Immune Cells (imInt)
KimInt_aEp = 0;
KimInt_imEp = 0;
KimInt_cEp = 0;
KimInt_cEpMito = 0;
KimInt_cEpLyso = 0;
KimInt_int = AimInt/VimInt*(Pn*fintN+Pd*NimInt/(exp(NimInt)-1)*fintD);
KimInt_sm = 0;
KimInt_imInt = AimInt/VimInt*(Pn*(-fimIntN)+Pd*NimInt/(exp(NimInt)-1)*(-fimIntD)*exp(NimInt));
KimInt_cEd = 0;
KimInt_cEdMito = 0;
KimInt_cEdLyso = 0;
KimInt_p = 0;
SimInt = 0;

% #9: Endothelial celss (cEd)
KcEd_aEp = 0;
KcEd_imEp = 0;
KcEd_cEp = 0;
KcEd_cEpMito = 0;
KcEd_cEpLyso = 0;
KcEd_int = -AbEd/VcEd*(Pn*(-fintN)+Pd*NbEd/(exp(NbEd)-1)*(-fintD)*exp(NbEd));
KcEd_sm = 0;
KcEd_imInt = 0;
KcEd_cEd = -AbEd/VcEd*(Pn*(fcEdN)+Pd*NbEd/(exp(NbEd)-1)*(fcEdD))...
        -AcEdMito/VcEd*(Pn*fcEdN+Pd*NcEdMito/(exp(NcEdMito)-1)*fcEdD)...
        -AcEdLyso/VcEd*(Pn*fcEdN+Pd*NcEdLyso/(exp(NcEdLyso)-1)*fcEdD)...
        +AaEd/VcEd*(Pn*(-fcEdN)+Pd*NaEd/(exp(NaEd)-1)*(-fcEdD)*exp(NaEd));
KcEd_cEdMito = -AcEdMito/VcEd*(Pn*(-fcEdMitoN)+Pd*NcEdMito/(exp(NcEdMito)-1)*(-
fcEdMitoD)*exp(NcEdMito));
KcEd_cEdLyso = -AcEdLyso/VcEd*(Pn*(-fcEdLysoN)+Pd*NcEdLyso/(exp(NcEdLyso)-1)*(-
fcEdLysoD)*exp(NcEdLyso));
KcEd_p = AaEd/VcEd*(Pn*(fpN)+Pd*NaEd/(exp(NaEd)-1)*(fpD));
ScEd = 0;


% #10: Endothelial celss (cEd) Mito
KcEdMito_aEp = 0;
KcEdMito_imEp = 0;
KcEdMito_cEp = 0;
KcEdMito_cEpMito = 0;
KcEdMito_cEpLyso = 0;
KcEdMito_int = 0;
KcEdMito_sm = 0;
KcEdMito_imInt = 0;
KcEdMito_cEd = AcEdMito/VcEdMito*(Pn*(fcEdN)+Pd*NcEdMito/(exp(NcEdMito)-1)*(fcEdD)) ;
KcEdMito_cEdMito = AcEdMito/VcEdMito*(Pn*(-fcEdMitoN)+Pd*NcEdMito/(exp(NcEdMito)-1)*(-
fcEdMitoD)*exp(NcEdMito));
KcEdMito_cEdLyso = 0;
KcEdMito_p = 0 ;
ScEdMito = 0;

% #11: Endothelial celss (cEd) Lyso
KcEdLyso_aEp = 0;
KcEdLyso_imEp = 0;
```

```
KcEdLyso_cEp = 0;
KcEdLyso_cEpMito = 0;
KcEdLyso_cEpLyso = 0;
KcEdLyso_int = 0 ;
KcEdLyso_sm = 0;
KcEdLyso_imInt = 0;
KcEdLyso_cEd = AcEdLyso/VcEdLyso*(Pn*(fcEdN)+Pd*NcEdLyso/(exp(NcEdLyso)-1)*(fcEdD)) ;
KcEdLyso_cEdMito = 0;
KcEdLyso_cEdLyso = AcEdLyso/VcEdLyso*(Pn*(-fcEdLysoN)+Pd*NcEdLyso/(exp(NcEdLyso)-1)*(-
fcEdLysoD)*exp(NcEdLyso));
KcEdLyso_p = 0;
ScEdLyso = 0;

% #12: plasma(p)
Kp_aEp = 0;
Kp_imEp = 0;
Kp_cEp = 0;
Kp_cEpMito = 0;
Kp_cEpLyso = 0;
Kp_int = 0;
Kp_sm = 0;
Kp_imInt = 0;
Kp_cEd = -AaEd/Vp*(Pn*(-fcEdN)+Pd*NaEd/(exp(NaEd)-1)*(-fcEdD)*exp(NaEd));
Kp_cEdMito = 0;
Kp_cEdLyso = 0;
Kp_p = -AaEd/Vp*(Pn*(fpN)+Pd*NaEd/(exp(NaEd)-1)*(fpD));
Sp = 0;

M =
[KaEp_aEp,KaEp_imEp,KaEp_cEp,KaEp_cEpMito,KaEp_cEpLyso,KaEp_int,KaEp_sm,KaEp_imInt,KaEp_cEd,K
aEp_cEdMito,KaEp_cEdLyso,KaEp_p;...

KimEp_aEp,KimEp_imEp,KimEp_cEp,KimEp_cEpMito,KimEp_cEpLyso,KimEp_int,KimEp_sm,KimEp_imInt,K
imEp_cEd,KimEp_cEdMito,KimEp_cEdLyso,KimEp_p;...

KcEp_aEp,KcEp_imEp,KcEp_cEp,KcEp_cEpMito,KcEp_cEpLyso,KcEp_int,KcEp_sm,KcEp_imInt,KcEp_cEd,Kc
Ep_cEdMito,KcEp_cEdLyso,KcEp_p;...

KcEpMito_aEp,KcEpMito_imEp,KcEpMito_cEp,KcEpMito_cEpMito,KcEpMito_cEpLyso,KcEpMito_int,KcEpMi
to_sm,KcEpMito_imInt,KcEpMito_cEd,KcEpMito_cEdMito,KcEpMito_cEdLyso,KcEpMito_p;...

KcEpLyso_aEp,KcEpLyso_imEp,KcEpLyso_cEp,KcEpLyso_cEpMito,KcEpLyso_cEpLyso,KcEpLyso_int,KcEpL
yso_sm,KcEpLyso_imInt,KcEpLyso_cEd,KcEpLyso_cEdMito,KcEpLyso_cEdLyso,KcEpLyso_p;...

Kint_aEp,Kint_imEp,Kint_cEp,Kint_cEpMito,Kint_cEpLyso,Kint_int,Kint_sm,Kint_imInt,Kint_cEd,Kint_cEdMit
o,Kint_cEdLyso,Kint_p;...

Ksm_aEp,Ksm_imEp,Ksm_cEp,Ksm_cEpMito,Ksm_cEpLyso,Ksm_int,Ksm_sm,Ksm_imInt,Ksm_cEd,Ksm_cEd
Mito,Ksm_cEdLyso,Ksm_p;...

KimInt_aEp,KimInt_imEp,KimInt_cEp,KimInt_cEpMito,KimInt_cEpLyso,KimInt_int,KimInt_sm,KimInt_imInt,K
imInt_cEd,KimInt_cEdMito,KimInt_cEdLyso,KimInt_p;...

KcEd_aEp,KcEd_imEp,KcEd_cEp,KcEd_cEpMito,KcEd_cEpLyso,KcEd_int,KcEd_sm,KcEd_imInt,KcEd_cEd,Kc
Ed_cEdMito,KcEd_cEdLyso,KcEd_p;...
```

```
KcEdMito_aEp,KcEdMito_imEp,KcEdMito_cEp,KcEdMito_cEpMito,KcEdMito_cEpLyso,KcEdMito_int,KcEdMito_sm,KcEdMito_imInt,KcEdMito_cEd,KcEdMito_cEdMito,KcEdMito_cEdLyso,KcEdMito_p;...

KcEdLyso_aEp,KcEdLyso_imEp,KcEdLyso_cEp,KcEdLyso_cEpMito,KcEdLyso_cEpLyso,KcEdLyso_int,KcEdLyso_sm,KcEdLyso_imInt,KcEdLyso_cEd,KcEdLyso_cEdMito,KcEdLyso_cEdLyso,KcEdLyso_p;...

Kp_aEp,Kp_imEp,Kp_cEp,Kp_cEpMito,Kp_cEpLyso,Kp_int,Kp_sm,Kp_imInt,Kp_cEd,Kp_cEdMito,Kp_cEdLyso,Kp_p];

G = [SaEp,SimEp,ScEp,ScEpMito,ScEpLyso,Sint,Ssm,SimInt,ScEd,ScEdMito,ScEdLyso,Sp]';
```

```
% By J.Yu @ 5/5/2010
% This is the generic PBPK model of atenolol in rat

% Virtual Lung (with Mito and Lyso in cEp and cEd) - PBPK
% Six big compartment model: arterial blood, lung, venous blood, liver, brain, and rest

function [dConc] = H_Lung_RL(t,Conc)
global V_VEN Vp fup B2P Vtot;
%call lung model
[LungM, LungG, M_v, Vp] = H_al_RL(); % get the coefficients for the Alveolar Region
[LungM_Airways, LungG_Airways, M_v_Airways, Vp_Airways] = H_aw_RL(); % get the coefficients for the
airways

% Body weight (kg)
BW = 0.25 ;

% From PATRICK POULIN, FRANK-PETER. THEILPrediction of Pharmacokinetics prior to In Vivo Studies.
% II. Generic Physiologically Based Pharmacokinetic Models of Drug Disposition
% Blood flow rate (mL/min)
Q_tot = 0.235*BW^0.75*1000 ; % Total cardiac output = 0.235 * body weight (kg)^0.75 (L/min)
Q_LUN = Q_tot ;
Q_HRT = 0.049*Q_tot ;
Q_BRA = 0.02*Q_tot ;
Q_LIV = 0.175*Q_tot ;
Q_GUT = 0.131*Q_tot ;
Q_KID = 0.141*Q_tot ;
Q_MUS = 0.278*Q_tot ;
Q_SKN = 0.058*Q_tot ;
Q_ADI = 0.07*Q_tot ;
Q_SPL = 0.02*Q_tot ;
Q_BON = 0.122*Q_tot ;
Q_Airways = 0.01*Q_tot;
Q_RES = Q_tot - Q_HRT - Q_BRA - Q_LIV - Q_KID - Q_MUS - Q_SKN - Q_ADI - Q_BON - Q_Airways ;


% From PATRICK POULIN, FRANK-PETER. THEILPrediction of Pharmacokinetics prior to In Vivo Studies.
% II. Generic Physiologically Based Pharmacokinetic Models of Drug Disposition
% Volume of each organ (mL)= fraction of total body volume (L/kg)*BW*1000
V_ART = 0.0272*BW*1000 ;
V_LUN = trace(M_v)*10^6; % total lung volume, in mL
V_LUNp = Vp*10^(6) ; % plasma volume in the lung, obtained from 'LungRatReverse' (in m^3), converted to mL
V_LUNb = 519*10^(-3)*V_LUN ; % total blood volume in the lung = 519uL/g
V_VEN = 0.0544*BW*1000 ;
V_HRT = 0.0033*BW*1000 ;
V_BRA = 0.0057*BW*1000 ;
V_LIV = 0.0366*BW*1000 ;
V_GUT = 0.027*BW*1000 ;
V_KID = 0.0073*BW*1000 ;
V_MUS = 0.404*BW*1000 ;
V_SKN = 0.19*BW*1000 ;
V_ADI = 0.076*BW*1000 ;
V_SPL = 0.002*BW*1000 ;
V_BON = 0.04148*BW*1000 ;
V_LUN_Airways = trace(M_v_Airways)*10^6 ;
V_LUNp_Airways = Vp_Airways*10^(6) ;
V_LUNb_Airways = 519*10^(-3)*V_LUN_Airways ;
```

```
V_RES = BW*1000-V_ART-V_LUN-V_LUNp-V_LUNb-V_LUN_Airways-V_LUNp_Airways-
V_LUNb_Airways-V_VEN - V_HRT...
       -V_BRA-V_LIV-V_GUT-V_KID-V_MUS-V_SKN-V_ADI-V_SPL-V_BON ;

B2P = 0.80 ;
fup = 0.13 ;
Kp_LUN = 54.90/B2P ;    % exp data
Kp_HRT = 4.97/B2P ;     % exp data
Kp_BRA = 9.20/B2P ;     % exp data
Kp_LIV = 5.67/B2P ;
Kp_GUT = 8.22/B2P ;
Kp_KID = 3.80/B2P ;     % exp data
Kp_MUS = 2.20/B2P ;     % exp data
Kp_SKN = 7.22/B2P ;
Kp_ADI = 0.18/B2P ;
Kp_SPL = 2.98/B2P ;
Kp_BON = 6.90/B2P ;
Kp_RES = 1/B2P ;

% Dosing IV information mg/kg, rats weight = 0.25kg
Kiv = 0 ;

% Mass balance
% 1 - Arterial, ART
% 2 - Lung alveoli plasma free concentration, LUN,
   % Cellular compartments of the lung alveoli:
   % 15 - Surface lining liquid (aEp)
   % 16 - Macrophage (imEp)
   % 17 - Epithelial cells (cEp)
   % 18 - Epithelial cells Mito (cEpMito)
   % 19 - Epithelial cells Lyso (cEpLyso)
   % 20 - Interstitium (int)
   % 21 - Smooth muscle (sm)
   % 22 - Immune cells (imInt)
   % 23 - Endothelial cells (cEd)
   % 24 - Endothelial cells Mito (cEdMito)
   % 25 - Endothelial cells Lyso (cEdLyso)
% 3 - Venous, VEN
% 4 - Heart, HRT
% 5 - Brain, BRA
% 6 - Liver, LIV, may be eliminatin organ
% 7 - Gut, GUT
% 8 - Spleen, SPL
% 9 - Kidney, KID, may be elimination organ
% 10 - Muscle, MUS
% 11 - Skin, SKN
% 12 - Adipose, ADI
% 13 - Bone, BON
% 14 - Rest of body, RES
% 26 - Lung airway plasma free concentration, LUN_airways
   % Cellular compartments of the lung airways:
   % 27 - Surface lining liquid (aEp_airway)
   % 28 - Macrophage (imEp_airway)
   % 29 - Epithelial cells (cEp_airway)
   % 30 - Epithelial cells Mito (cEpMito_airway)
```

```
   % 31 - Epithelial cells Lyso (cEpLyso_airway)
   % 32 - Interstitium (int_airway)
   % 33 - Smooth muscle (sm_airway)
   % 34 - Smooth muscle Mito (smMito_airway)
   % 35 - Smooth muscle Lyso (smLyso_airway)
   % 36 - Immune cells (imInt_airway)
   % 37 - Endothelial cells (cEd_airway)
   % 38 - Endothelial cells Mito (cEdMito_airway)
   % 39 - Endothelial cells Lyso (cEdLyso_airway)


% fup: fraction of unbound in the plamsa
% Kp: Tissue:Blood partition coefficients = C_tissue, tot : C_blood, tot

dConc(1) = Conc(2)*Q_tot/V_ART - Conc(1)*Q_tot/V_ART ; % ART, arterial blood

dConc(2) = Conc(3)*Q_tot/V_LUNb + (LungM(12,1)*Conc(15) + LungM(12,2)*Conc(16) +
LungM(12,3)*Conc(17) + LungM(12,4)*Conc(18)...
        + LungM(12,5)*Conc(19) + LungM(12,6)*Conc(21) + LungM(12,7)*Conc(21) + LungM(12,8)*Conc(22) +
LungM(12,9)*Conc(23) ...
        + LungM(12,10)*Conc(24) + LungM(12,11)*Conc(25) + LungM(12,12)*Conc(2)*fup/B2P +
LungG(12))*V_LUNp/V_LUNb - Conc(2)*Q_tot/V_LUNb ;
                                                    % 2 -Lung total blood concentration,C(2)*fup/B2P = free plasma conc in the
lung alveoli

   dConc(15) = LungM(1,1)*Conc(15) + LungM(1,2)*Conc(16) + LungM(1,3)*Conc(17) + LungM(1,4)*Conc(18)
+ LungM(1,5)*Conc(19)...
            + LungM(1,6)*Conc(20) + LungM(1,7)*Conc(21) + LungM(1,8)*Conc(22) + LungM(1,9)*Conc(23) +
LungM(1,10)*Conc(24)...
            + LungM(1,11)*Conc(25) + LungM(1,12)*Conc(2)*fup/B2P + LungG(1);                              % 15 -
SurfaConce lining liquid (aEp)

   dConc(16) = LungM(2,1)*Conc(15) + LungM(2,2)*Conc(16) + LungM(2,3)*Conc(17) + LungM(2,4)*Conc(18)
+ LungM(2,5)*Conc(19)...
            + LungM(2,6)*Conc(20) + LungM(2,7)*Conc(21) + LungM(2,8)*Conc(22) + LungM(2,9)*Conc(23) +
LungM(2,10)*Conc(24)...
            + LungM(2,11)*Conc(25) + LungM(2,12)*Conc(2)*fup/B2P + LungG(2);                              % 16 -
Macrophage (imEp)

   dConc(17) = LungM(3,1)*Conc(15) + LungM(3,2)*Conc(16) + LungM(3,3)*Conc(17) + LungM(3,4)*Conc(18)
+ LungM(3,5)*Conc(19)...
            + LungM(3,6)*Conc(20) + LungM(3,7)*Conc(21) + LungM(3,8)*Conc(22) + LungM(3,9)*Conc(23) +
LungM(3,10)*Conc(24)...
            + LungM(3,11)*Conc(25) +LungM(3,12)*Conc(2)*fup/B2P + LungG(3);                               % 17 -
Epithelial cells (cEp)

   dConc(18) = LungM(4,1)*Conc(15) + LungM(4,2)*Conc(16) + LungM(4,3)*Conc(17) + LungM(4,4)*Conc(18)
+ LungM(4,5)*Conc(19)...
            + LungM(4,6)*Conc(20) + LungM(4,7)*Conc(21) + LungM(4,8)*Conc(22) + LungM(4,9)*Conc(23) +
LungM(4,10)*Conc(24)...
            + LungM(4,11)*Conc(25) + LungM(4,12)*Conc(2)*fup/B2P + LungG(4);                              % 18 -
Epithelial cells (cEpMito)

   dConc(19) = LungM(5,1)*Conc(15) + LungM(5,2)*Conc(16) + LungM(5,3)*Conc(17) + LungM(5,4)*Conc(18)
+ LungM(5,5)*Conc(19)...
```

```
            + LungM(5,6)*Conc(20) + LungM(5,7)*Conc(21) + LungM(5,8)*Conc(22) + LungM(5,9)*Conc(23) +
LungM(5,10)*Conc(24)...
            + LungM(5,11)*Conc(25) + LungM(5,12)*Conc(2)*fup/B2P + LungG(5);                             % 19 -
Epithelial cells (cEpLyso)

    dConc(20) = LungM(6,1)*Conc(15) + LungM(6,2)*Conc(16) + LungM(6,3)*Conc(17) + LungM(6,4)*Conc(18)
+ LungM(6,5)*Conc(19)...
            + LungM(6,6)*Conc(20) + LungM(6,7)*Conc(21) + LungM(6,8)*Conc(22) + LungM(6,9)*Conc(23) +
LungM(6,10)*Conc(24)...
            + LungM(6,11)*Conc(25) + LungM(6,12)*Conc(2)*fup/B2P + LungG(6);                             % 20 -
Interstitium (int)

    dConc(21) = LungM(7,1)*Conc(15) + LungM(7,2)*Conc(16) + LungM(7,3)*Conc(17) + LungM(7,4)*Conc(18)
+ LungM(7,5)*Conc(19)...
            + LungM(7,6)*Conc(20) + LungM(7,7)*Conc(21) + LungM(7,8)*Conc(22) + LungM(7,9)*Conc(23) +
LungM(7,10)*Conc(24)...
            + LungM(7,11)*Conc(25) + LungM(7,12)*Conc(2)*fup/B2P + LungG(7);                             % 21 -
Smooth muscle (sm)


    dConc(22) = LungM(8,1)*Conc(15) + LungM(8,2)*Conc(16) + LungM(8,3)*Conc(17) + LungM(8,4)*Conc(18)
+ LungM(8,5)*Conc(19)...
            + LungM(8,6)*Conc(20) + LungM(8,7)*Conc(21) + LungM(8,8)*Conc(22) + LungM(8,9)*Conc(23) +
LungM(8,10)*Conc(24)...
            + LungM(8,11)*Conc(25) + LungM(8,12)*Conc(2)*fup/B2P + LungG(8);                             % 22 -
Immune cells (imInt)

    dConc(23) = LungM(9,1)*Conc(15) + LungM(9,2)*Conc(16) + LungM(9,3)*Conc(17) + LungM(9,4)*Conc(18)
+ LungM(9,5)*Conc(19)...
            + LungM(9,6)*Conc(20) + LungM(9,7)*Conc(21) + LungM(9,8)*Conc(22) + LungM(9,9)*Conc(23) +
LungM(9,10)*Conc(24)...
            + LungM(9,11)*Conc(25) + LungM(9,12)*Conc(2)*fup/B2P + LungG(9);                             % 23 -
Endothelial cells (cEd)

    dConc(24) = LungM(10,1)*Conc(15) + LungM(10,2)*Conc(16) + LungM(10,3)*Conc(17) +
LungM(10,4)*Conc(18) + LungM(10,5)*Conc(19)...
            + LungM(10,6)*Conc(20) + LungM(10,7)*Conc(21) + LungM(10,8)*Conc(22) + LungM(10,9)*Conc(23)
+ LungM(10,10)*Conc(24)...
            + LungM(10,11)*Conc(25) + LungM(10,12)*Conc(2)*fup/B2P + LungG(10);                             % 24 -
Endothelial cells (cEdMito)

    dConc(25) = LungM(11,1)*Conc(15) + LungM(11,2)*Conc(16) + LungM(11,3)*Conc(17) +
LungM(11,4)*Conc(18) + LungM(11,5)*Conc(19)...
            + LungM(11,6)*Conc(20) + LungM(11,7)*Conc(21) + LungM(11,8)*Conc(22) + LungM(11,9)*Conc(23)
+ LungM(11,10)*Conc(24)...
            + LungM(11,11)*Conc(25) + LungM(11,12)*Conc(2)*fup/B2P + LungG(11);                             % 25 -
Endothelial cells (cEdLyso)


dConc(3) = Conc(4)*Q_HRT/Kp_HRT/V_VEN + Conc(5)*Q_BRA/Kp_BRA/V_VEN +
Conc(6)*Q_LIV/Kp_LIV/V_VEN...
        + Conc(9)*Q_KID/Kp_KID/V_VEN + Conc(10)*Q_MUS/Kp_MUS/V_VEN +
Conc(11)*Q_SKN/Kp_SKN/V_VEN...
        + Conc(12)*Q_ADI/Kp_ADI/V_VEN + Conc(13)*Q_BON/Kp_BON/V_VEN +
Conc(14)*Q_RES/Kp_RES/V_VEN...
        + Conc(26)*Q_Airways/V_VEN - Conc(3)*Q_tot/V_VEN + Kiv/V_VEN ;      % Venous blood
```

```
dConc(4) = Conc(1)*Q_HRT/V_HRT - Conc(4)*Q_HRT/Kp_HRT/V_HRT ;                    % Heart
dConc(5) = Conc(1)*Q_BRA/V_BRA - Conc(5)*Q_BRA/Kp_BRA/V_BRA ;                    % Brain

% If consider the extraction ratio, per definition E = (Ca-Cv)/Ca
% equation comes from: PATRICK POULIN, FRANK-PETER. THEILPrediction of Pharmacokinetics prior to In Vivo Studies.
% II. Generic Physiologically Based Pharmacokinetic Models of Drug Disposition
E_LIV = 0;
dConc(6) = (((Q_LIV-Q_GUT-Q_SPL)*Conc(1)+(Q_GUT*Conc(7)/Kp_GUT + Q_SPL*Conc(8)/Kp_SPL - Q_LIV*Conc(6)/Kp_LIV))/V_LIV)...
    - ((((Q_LIV-Q_GUT-Q_SPL)*Conc(1)+(Q_GUT*Conc(7)/Kp_GUT + Q_SPL*Conc(8)/Kp_SPL))*E_LIV))/V_LIV ;

dConc(7) = Conc(1)*Q_GUT/V_GUT - Conc(7)*Q_GUT/Kp_GUT/V_GUT ;                    % Gut
dConc(8) = Conc(1)*Q_SPL/V_SPL - Conc(8)*Q_SPL/Kp_SPL/V_SPL ;                    % Spleen
dConc(9) = Conc(1)*Q_KID/V_KID - Conc(9)*Q_KID/Kp_KID/V_KID ;                    % Kidney
dConc(10) = Conc(1)*Q_MUS/V_MUS - Conc(10)*Q_MUS/Kp_MUS/V_MUS ;                  % Muscle
dConc(11) = Conc(1)*Q_SKN/V_SKN - Conc(11)*Q_SKN/Kp_SKN/V_SKN ;                  % Skin
dConc(12) = Conc(1)*Q_ADI/V_ADI - Conc(12)*Q_ADI/Kp_ADI/V_ADI ;                  % Adipose
dConc(13) = Conc(1)*Q_BON/V_BON - Conc(13)*Q_BON/Kp_BON/V_BON ;                  % Bone
dConc(14) = Conc(1)*Q_RES/V_RES - Conc(14)*Q_RES/Kp_RES/V_RES ;                  % Rest of body


dConc(26) = Conc(1)*Q_Airways/V_LUNb_Airways - Conc(26)*Q_Airways/V_LUNb_Airways...   % 26 -Lung airways blood concentration, C(2)*fup/B2P = free plasma conc
        + (LungM_Airways(14,1)*Conc(27) + LungM_Airways(14,2)*Conc(28) + LungM_Airways(14,3)*Conc(29) + LungM_Airways(14,4)*Conc(30)...
        + LungM_Airways(14,5)*Conc(31) + LungM_Airways(14,6)*Conc(24) + LungM_Airways(14,7)*Conc(33) + LungM_Airways(14,8)*Conc(34)...
        + LungM_Airways(14,9)*Conc(35) + LungM_Airways(14,10)*Conc(36) + LungM_Airways(14,11)*Conc(37) + LungM_Airways(14,12)*Conc(38)...
        + LungM_Airways(14,13)*Conc(39) + LungM_Airways(14,14)*Conc(26)*fup/B2P + LungG_Airways(14))*V_LUNp_Airways/V_LUNb_Airways;  % 26 -Lung airways blood concentration, C(2)*fup/B2P = free plasma conc



  dConc(27) = LungM_Airways(1,1)*Conc(27) + LungM_Airways(1,2)*Conc(28) + LungM_Airways(1,3)*Conc(29) + LungM_Airways(1,4)*Conc(30) + LungM_Airways(1,5)*Conc(31)...
        + LungM_Airways(1,6)*Conc(32) + LungM_Airways(1,7)*Conc(33) + LungM_Airways(1,8)*Conc(34) + LungM_Airways(1,9)*Conc(35) + LungM_Airways(1,10)*Conc(36)...
        + LungM_Airways(1,11)*Conc(37)+ LungM_Airways(1,12)*Conc(38)+ LungM_Airways(1,13)*Conc(39) ...
        + LungM_Airways(1,14)*Conc(26)*fup/B2P + LungG_Airways(1);               % 27 - Airways SurfaConce lining liquid (aEp)

  dConc(28) = LungM_Airways(2,1)*Conc(27) + LungM_Airways(2,2)*Conc(28) + LungM_Airways(2,3)*Conc(29) + LungM_Airways(2,4)*Conc(30) + LungM_Airways(2,5)*Conc(31)...
        + LungM_Airways(2,6)*Conc(32) + LungM_Airways(2,7)*Conc(33) + LungM_Airways(2,8)*Conc(34) + LungM_Airways(2,9)*Conc(35) + LungM_Airways(2,10)*Conc(36)...
```

```
            + LungM_Airways(2,11)*Conc(37)+ LungM_Airways(2,12)*Conc(38)+
LungM_Airways(2,13)*Conc(39) ...
            + LungM_Airways(2,14)*Conc(26)*fup/B2P + LungG_Airways(2);                    % 28 - Airways
Macrophage (imEp)

    dConc(29) = LungM_Airways(3,1)*Conc(27) + LungM_Airways(3,2)*Conc(28) +
LungM_Airways(3,3)*Conc(29) + LungM_Airways(3,4)*Conc(30) + LungM_Airways(3,5)*Conc(31)...
            + LungM_Airways(3,6)*Conc(32) + LungM_Airways(3,7)*Conc(33) + LungM_Airways(3,8)*Conc(34) +
LungM_Airways(3,9)*Conc(35) + LungM_Airways(3,10)*Conc(36)...
            + LungM_Airways(3,11)*Conc(37)+ LungM_Airways(3,12)*Conc(38)+
LungM_Airways(3,13)*Conc(39) ...
            +LungM_Airways(3,14)*Conc(26)*fup/B2P + LungG_Airways(3);                     % 29 - Airways
Epithelial cells (cEp)

    dConc(30) = LungM_Airways(4,1)*Conc(27) + LungM_Airways(4,2)*Conc(28) +
LungM_Airways(4,3)*Conc(29) + LungM_Airways(4,4)*Conc(30) + LungM_Airways(4,5)*Conc(31)...
            + LungM_Airways(4,6)*Conc(32) + LungM_Airways(4,7)*Conc(33) + LungM_Airways(4,8)*Conc(34) +
LungM_Airways(4,9)*Conc(35) + LungM_Airways(4,10)*Conc(36)...
            + LungM_Airways(4,11)*Conc(37)+ LungM_Airways(4,12)*Conc(38)+
LungM_Airways(4,13)*Conc(39) ...
            + LungM_Airways(4,14)*Conc(26)*fup/B2P + LungG_Airways(4);                    % 30 - Airways
Epithelial cells (cEpMito)

    dConc(31) = LungM_Airways(5,1)*Conc(27) + LungM_Airways(5,2)*Conc(28) +
LungM_Airways(5,3)*Conc(29) + LungM_Airways(5,4)*Conc(30) + LungM_Airways(5,5)*Conc(31)...
            + LungM_Airways(5,6)*Conc(32) + LungM_Airways(5,7)*Conc(33) + LungM_Airways(5,8)*Conc(34) +
LungM_Airways(5,9)*Conc(35) + LungM_Airways(5,10)*Conc(36)...
            + LungM_Airways(5,11)*Conc(37)+ LungM_Airways(5,12)*Conc(38)+
LungM_Airways(5,13)*Conc(39) ...
            + LungM_Airways(5,14)*Conc(26)*fup/B2P + LungG_Airways(5);                    % 31 - Airways
Epithelial cells (cEpLyso)

    dConc(32) = LungM_Airways(6,1)*Conc(27) + LungM_Airways(6,2)*Conc(28) +
LungM_Airways(6,3)*Conc(29) + LungM_Airways(6,4)*Conc(30) + LungM_Airways(6,5)*Conc(31)...
            + LungM_Airways(6,6)*Conc(32) + LungM_Airways(6,7)*Conc(33) + LungM_Airways(6,8)*Conc(34) +
LungM_Airways(6,9)*Conc(35) + LungM_Airways(6,10)*Conc(36)...
            + LungM_Airways(6,11)*Conc(37)+ LungM_Airways(6,12)*Conc(38)+
LungM_Airways(6,13)*Conc(39) ...
            + LungM_Airways(6,14)*Conc(26)*fup/B2P + LungG_Airways(6);                    % 32 - Airways
Interstitium (int)

    dConc(33) = LungM_Airways(7,1)*Conc(27) + LungM_Airways(7,2)*Conc(28) +
LungM_Airways(7,3)*Conc(29) + LungM_Airways(7,4)*Conc(30) + LungM_Airways(7,5)*Conc(31)...
            + LungM_Airways(7,6)*Conc(32) + LungM_Airways(7,7)*Conc(33) + LungM_Airways(7,8)*Conc(34) +
LungM_Airways(7,9)*Conc(35) + LungM_Airways(7,10)*Conc(36)...
            + LungM_Airways(7,14)*Conc(37)+ LungM_Airways(7,12)*Conc(38)+
LungM_Airways(7,13)*Conc(39) ...
            + LungM_Airways(7,12)*Conc(26)*fup/B2P + LungG_Airways(7);                    % 33 - Airways
Smooth muscle (sm)


    dConc(34) = LungM_Airways(8,1)*Conc(27) + LungM_Airways(8,2)*Conc(28) +
LungM_Airways(8,3)*Conc(29) + LungM_Airways(8,4)*Conc(30) + LungM_Airways(8,5)*Conc(31)...
            + LungM_Airways(8,6)*Conc(32) + LungM_Airways(8,7)*Conc(33) + LungM_Airways(8,8)*Conc(34) +
LungM_Airways(8,9)*Conc(35) + LungM_Airways(8,10)*Conc(36)...
```

```
            + LungM_Airways(8,11)*Conc(37)+ LungM_Airways(8,12)*Conc(38)+
LungM_Airways(8,13)*Conc(39) ...
            + LungM_Airways(8,14)*Conc(26)*fup/B2P + LungG_Airways(8);                    % 34 - Airways
Smooth muscle (smMito)

    dConc(35) = LungM_Airways(9,1)*Conc(27) + LungM_Airways(9,2)*Conc(28) +
LungM_Airways(9,3)*Conc(29) + LungM_Airways(9,4)*Conc(30) + LungM_Airways(9,5)*Conc(31)...
            + LungM_Airways(9,6)*Conc(32) + LungM_Airways(9,7)*Conc(33) + LungM_Airways(9,8)*Conc(34) +
LungM_Airways(9,9)*Conc(35) + LungM_Airways(9,10)*Conc(36)...
            + LungM_Airways(9,11)*Conc(37)+ LungM_Airways(9,12)*Conc(38)+
LungM_Airways(9,13)*Conc(39) ...
            + LungM_Airways(9,14)*Conc(26)*fup/B2P + LungG_Airways(9);                    % 35 - Airways
Smooth muscle (smLyso)

    dConc(36) = LungM_Airways(10,1)*Conc(27) + LungM_Airways(10,2)*Conc(28) +
LungM_Airways(10,3)*Conc(29) + LungM_Airways(10,4)*Conc(30) + LungM_Airways(10,5)*Conc(31)...
            + LungM_Airways(10,6)*Conc(32) + LungM_Airways(10,7)*Conc(33) +
LungM_Airways(10,8)*Conc(34) + LungM_Airways(10,9)*Conc(35) + LungM_Airways(10,10)*Conc(36)...
            + LungM_Airways(10,11)*Conc(37)+ LungM_Airways(10,12)*Conc(38)+
LungM_Airways(10,13)*Conc(39) ...
            + LungM_Airways(10,14)*Conc(26)*fup/B2P + LungG_Airways(10);                  % 36 - Airways
Immune cells (imInt)

    dConc(37) = LungM_Airways(11,1)*Conc(27) + LungM_Airways(11,2)*Conc(28) +
LungM_Airways(11,3)*Conc(29) + LungM_Airways(11,4)*Conc(30) + LungM_Airways(11,5)*Conc(31)...
            + LungM_Airways(11,6)*Conc(32) + LungM_Airways(11,7)*Conc(33) +
LungM_Airways(11,8)*Conc(34) + LungM_Airways(11,9)*Conc(35) + LungM_Airways(11,10)*Conc(36)...
            + LungM_Airways(11,11)*Conc(37)+ LungM_Airways(11,12)*Conc(38)+
LungM_Airways(11,13)*Conc(39)  ...
            + LungM_Airways(11,14)*Conc(26)*fup/B2P + LungG_Airways(11);                  % 37 - Airways
Endothelial cells

    dConc(38) = LungM_Airways(12,1)*Conc(27) + LungM_Airways(12,2)*Conc(28) +
LungM_Airways(12,3)*Conc(29) + LungM_Airways(12,4)*Conc(30) + LungM_Airways(12,5)*Conc(31)...
            + LungM_Airways(12,6)*Conc(32) + LungM_Airways(12,7)*Conc(33) +
LungM_Airways(12,8)*Conc(34) + LungM_Airways(12,9)*Conc(35) + LungM_Airways(12,10)*Conc(36)...
            + LungM_Airways(12,11)*Conc(37)+ LungM_Airways(12,12)*Conc(38)+
LungM_Airways(12,13)*Conc(39) ...
            + LungM_Airways(12,14)*Conc(26)*fup/B2P + LungG_Airways(12);                  % 38 -
Endothelial cells (cEdMito)

    dConc(39) = LungM_Airways(13,1)*Conc(27) + LungM_Airways(13,2)*Conc(28) +
LungM_Airways(13,3)*Conc(29) + LungM_Airways(13,4)*Conc(30) + LungM_Airways(13,5)*Conc(31)...
            + LungM_Airways(13,6)*Conc(32) + LungM_Airways(13,7)*Conc(33) +
LungM_Airways(13,8)*Conc(34) + LungM_Airways(13,9)*Conc(35) + LungM_Airways(13,10)*Conc(36)...
            + LungM_Airways(13,11)*Conc(37)+ LungM_Airways(13,12)*Conc(38)+
LungM_Airways(13,13)*Conc(39) ...
            + LungM_Airways(13,14)*Conc(26)*fup/B2P + LungG_Airways(13);                  % 39 -
Endothelial cells (cEdLyso)


Vtot = diag([V_ART V_LUNb V_VEN V_HRT V_BRA V_LIV V_GUT V_SPL V_KID....
           V_MUS V_SKN V_ADI V_BON V_RES diag(M_v)'*10^6 V_LUNb_Airways
diag(M_v_Airways)'*10^6]) ;
```

```
dConc = [dConc(1), dConc(2), dConc(3), dConc(4), dConc(5), dConc(6), dConc(7), dConc(8), dConc(9), dConc(10),...
    dConc(11), dConc(12), dConc(13), dConc(14), dConc(15), dConc(16), dConc(17), dConc(18), dConc(19), dConc(20),...
    dConc(21), dConc(22), dConc(23), dConc(24), dConc(25), dConc(26), dConc(27), dConc(28), dConc(29), dConc(30),...
    dConc(31), dConc(32), dConc(33), dConc(34), dConc(35), dConc(36), dConc(37), dConc(38), dConc(39)]' ;
```

```
% Run simulation with single bolus IV
% compartments and corresponding number

% function
function [T,Y,Conc_LUNSim,Con_al,Con_aw,Mass_LUNtemp,Mass_Airwaystemp]= H_RL_IH_fun()
global BW V_VEN fup B2P Vtot;


% calculate the concentration accumulated in the lung by Jerry's model
[M, G, M_v, Vp] = H_al_RL();
[M_Airways, G_Airways, M_v_Airways, Vp_Airways] = H_aw_RL();

BW = 0.25 ;
V_VEN = 0.0544*BW*1000 ;
Y0 = zeros(39,1) ;

%IV dose
Y0(3) = 1*10^6*(M_v(1,1)*10^6+M_v_Airways(1,1)*10^6)/V_VEN;

Yopt = 1e-13 * ones(1,39) ;
options = odeset('RelTol',1e-13,'AbsTol',Yopt);
[T,Y] = ode15s(@H_Lung_RL,[0 10*60],Y0,options);
len = length(T) ;
Conc_LUNtemp = Y(:,15:25) ;
Mass_LUNtemp = Conc_LUNtemp*(M_v*10^6) ; %ng,alveo

Conc_Airwaystemp = Y(:,27:39) ;
Mass_Airwaystemp = Conc_Airwaystemp*(M_v_Airways*10^6) ; %ng

Mass_LUNSim = sum(Mass_LUNtemp, 2) + sum(Mass_Airwaystemp, 2) ;%ng
Conc_LUNSim = Mass_LUNSim / ((trace(M_v)*10^6)+(trace(M_v_Airways)*10^6)); %ng/mL

Con_al = sum(Mass_LUNtemp, 2)/(trace(M_v)*10^6);%ng/ml
Con_aw = sum(Mass_Airwaystemp, 2)/(trace(M_v_Airways)*10^6);%ng/ml

% lung:venous blood concentration ratio (Kp_LUN)
Kpulung = Conc_LUNSim(len)/(Y(len,3)*fup/B2P) ;
Kplung = Conc_LUNSim(len)/Y(len,3) ;
Conc_LUN_1hr = Conc_LUNSim(len);%ng/ml
Conc_ven_1hr = Y(len,3);%ng/ml

% organelle mass/concentration
Mass = Y*Vtot;%ng
%mito
Mass_mito = Mass(:,18) + Mass(:,24) + Mass(:,30) + Mass(:,34) + Mass(:,38);
V_mito = Vtot(18,18) + Vtot(24,24) + Vtot(30,30) + Vtot(34,34) + Vtot(38,38);
C_mito = Mass_mito/V_mito;
%lyso
Mass_lyso = Mass(:,19) + Mass(:,25) + Mass(:,31) + Mass(:,35) + Mass(:,39);
V_lyso = Vtot(19,19) + Vtot(25,25) + Vtot(31,31) + Vtot(35,35) + Vtot(39,39);
C_lyso = Mass_lyso/V_lyso;
%cyto
Mass_cyto = Mass(:,16) + Mass(:,17) + Mass(:,21) + Mass(:,22) + Mass(:,23)...
    + Mass(:,28) + Mass(:,29) + Mass(:,33) + Mass(:,36) + Mass(:,37);
V_cyto = Vtot(16,16) + Vtot(17,17) + Vtot(21,21) + Vtot(22,22) + Vtot(23,23)...
    + Vtot(28,28) + Vtot(29,29) + Vtot(33,33) + Vtot(36,36) + Vtot(37,37);
```

```
C_cyto = Mass_cyto/V_cyto;

end
```

```
%Main function
clear
close all
clc

% ----------------------------------------------------------
% HOE 33324
%-----------------------------------------------------------
[HIH.T,HIH.Y,HIH.C_lung,HIH.C_al,HIH.C_aw]=H_RL_IH_fun();
AUC_al = trapz(HIH.T,HIH.C_al);
AUC_aw = trapz(HIH.T,HIH.C_aw);
HIH.L_al = num2str(AUC_al,'%6.2e');
HIH.L_aw = num2str(AUC_aw,'%6.2e');
% HIH.Y = HIH.Y.*10^6;
save('3','HIH');


Fig1 = figure;
sub1 = subplot(1,1,1,'Parent',Fig1,'YScale','log','YMinorTick','on',...
    'FontSize',30);
xlim(sub1,[0 60]);
% Uncomment the following line to preserve the Y-limits of the axes
ylim(sub1,[1 10^8]);
hold(sub1,'all');

for i = [3]
load(num2str(i));
AL = strcat('IV');
AW = AL;
plot(HIH.T,HIH.C_al,'DisplayName',strcat('Alveoli Total','; ', AL),'LineWidth',3);%Inhale,al
plot(HIH.T,HIH.C_aw,'DisplayName',strcat('Airways Total','; ', AW),'LineWidth',3);%Inhale,aw

% Create xlabel
xlabel('Time (min)','FontSize',30);
% Create ylabel
ylabel('Concentration (ng/ml)','FontSize',30);
title('HOE','FontSize',30);
legend1 = legend(sub1,'show');
set(legend1,'FontSize',24,'FontName','Arial');
end

hold off
```
