# Supplementary material for: A Cell-based Computational Modeling Approach for Developing Site-Directed Molecular Probes
Source: PLoS Comput Biol. 2012 Feb 23;8(2):e1002378. doi: 10.1371/journal.pcbi.1002378 (PMC3285574; doi:10.1371/journal.pcbi.1002378)
Supplement: Text S7 — Supplementary parameter values and sensitivity analysis. This file includes information detailing: 1) Constant input parameter values used in the simulations; 2) Parameter values and sensitivity analysis of AUC (mg/ml*min) for alveoli; 3) Parameter values and sensitivity analysis of AUC (mg/ml*min) for airways; 4) Parameter values and sensitivity analysis of the time to reach steady state (Tss, in min) for alveoli; 5) Parameter values and sensitivity analysis of the time to reach steady state (Tss, in min) for airways; 6) Parameter values and sensitivity analysis of mass deposition for alveoli as a fraction of the total mass in the lung; 7) Parameter values and sensitivity analysis of mass deposition in airways as a fraction of the total mass in the lung. (PDF) [file pcbi.1002378.s007.pdf]

Supplementary Information for

A Cell-based Computational Modeling Approach for Developing Site-Directed Molecular Probes

Jing-yu Yu[1#], Nan Zheng[1#], Gerta Mane[2], Kyoung Ah Min[1], Juan Hinestroza[3], Huaning Zhu[3], Kathleen A. Stringer[2], and Gus R. Rosania[1*.]

Table S1.

Parameters and Sensitivity Analysis

Table. I. Constant Parameters in the Model

Symbol	Value	Unit	description
T	310.15	K	Body temperature
R	8.314	J / mol / K	Universal gas constant
F	96485.3415	sA / mol	Faraday constant

Parameter abbreviations in Supplementary Tables: L indicates the volumetric fraction of lipids (dimensionless), G indicates the activity coefficient γ (dimensionless), A indicates the surface area of the membrane (unit: m^2), *V* indicates the volume of the corresponding compartment (unit: m^3). E indicates the membrane potential (unit: V), pH indicates the pH values in corresponding compartment (dimensionless). Ro indicates the volumetric percentage of organelles in cellular compartments (dimensionless). The subscripts *aEp* indicates the apical side of epithelial cells, *cEp* indicates the cytosol of epithelial, *imEp* indicates the macrophage/immune cells on the surface of epithelium, *int* indicates the interstitium, *imInt* indicates the immune cells in the interstitium, *sm*

indicates the smooth muscle, *cEd* indicates the cytosol of endothelium, *p* indicates the plasma.

Table. S2. Parameter values and sensitivity analysis of AUC (mg/ml*min) for alveoli

Parameter	Default	Low	High	Mean	SD	CV	Trend
LaEp	0.95	0.05	0.95	0.06148	0.01861	0.3027	+
LimEp	0.05	0.005	0.5	0.09819	0.001462	0.01489	+
LcEp	0.05	0.005	0.5	0.09709	0.000794	0.008183	+
Lint	0	0.005	0.5	0.09702	0.00055	0.005668	+
LimInt	0.05	0.005	0.5	0.09621	5.61E-05	0.000583	+
LcEd	0.05	0.005	0.5	0.09863	0.001656	0.0168	+
Lp	0	0.005	0.5	0.09612	6.08E-12	6.33E-11	-
GaEpN	1	0.5	1.5	0.09677	0.005074	0.05243	-
GaEpD	1	0.5	1.5	0.1017	0.02376	0.2336	-
GimEpN	1.23	0.5	1.5	0.09614	1.45E-05	0.000151	-
GimEpD	0.74	0.5	1.5	0.09569	0.000605	0.006326	-
GcEpN	1.23	0.5	1.5	0.09613	7.49E-06	7.79E-05	-
GcEpD	0.74	0.5	1.5	0.09589	0.000346	0.003612	-
GintN	1	0.5	1.5	0.09612	6.30E-07	6.55E-06	-
GintD	1	0.5	1.5	0.09615	0.000236	0.002452	-
GimIntN	1.23	0.5	1.5	0.09612	6.24E-07	6.50E-06	-
GimIntD	0.74	0.5	1.5	0.0961	2.73E-05	0.000284	-
GcEdN	1.23	0.5	1.5	0.09613	1.86E-05	0.000194	-
GcEdD	0.74	0.5	1.5	0.09553	0.000717	0.007507	-
GpN	1	0.5	1.5	0.09612	6.88E-13	7.16E-12	+
GpD	1	0.5	1.5	0.09612	1.12E-11	1.17E-10	+

AaEp	0.387	0.0387	3.87	0.06819	0.04024	0.5901	-
Aabp	0.387	0.0387	3.87	0.09149	0.01539	0.1682	-
AimEp	0.0042	0.00042	0.042	0.101	0.003111	0.0308	+
AimInt	0.00042	0.000042	0.0042	0.09612	1.32E-09	1.37E-08	+
AbEd	0.452	0.0452	4.52	0.09261	0.008726	0.09422	-
AaEd	0.452	0.0452	4.52	0.09281	0.01073	0.1156	-
ASL	5	0.5	50	0.1065	0.01065	0.1001	+
Vint	2.68E-07	2.68E-08	2.68E-06	0.07264	0.01426	0.1963	-
Ro	0.1	0.01	0.5	0.09777	0.001412	0.01444	+
pHaEp	7.4	4	9	0.09607	0.003608	0.03756	+
pHimEp	7	4	9	0.09593	0.001094	0.01141	-
pHcEp	7	4	9	0.09889	0.005637	0.057	+
pHint	7	4	9	0.1004	0.008185	0.08152	+
pHimInt	7	4	9	0.09612	4.42E-05	0.00046	-
pHcEd	7	4	9	0.09596	0.002246	0.02341	-
pHp	7.4	4	9	0.09612	1.28E-11	1.33E-10	-
EbEd	-0.06	-0.12	0	0.09933	0.01645	0.1656	+
EaEd	-0.06	-0.12	0	0.09759	0.009574	0.0981	-
EimInt	-0.06	-0.12	0	0.09616	0.000112	0.001164	-
EimEp	-0.06	-0.12	0	0.09388	8.63E-05	0.000919	+
EbEp	0.0119	0	0.12	0.07161	0.01334	0.1863	-
EaEp	-0.0093	-0.12	0	0.04605	0.02446	0.1311	+

Table. S3 Parameter values and sensitivity analysis of AUC (mg/ml*min) for airways

Parameter	Default	Low	High	Mean	SD	CV	Trend
LaEp	0.2	0.05	0.95	8.555	0.8889	0.1039	+
LcEp	0.05	0.005	0.5	7.83	0.3157	0.04032	+
Lint	0	0.005	0.5	7.509	0.03813	0.005078	+
LimInt	0.05	0.005	0.5	7.452	0.003692	0.000495	+
Lsm	0.05	0.005	0.5	9.912	1.706	0.1721	+
LcEd	0.05	0.005	0.5	7.47	0.01523	0.002039	+
Lp	0	0.005	0.5	7.447	1.18E-08	1.58E-09	-
GaEpN	1	0.5	1.5	7.453	0.05194	0.006969	-
GaEpD	1	0.5	1.5	7.54	0.3954	0.05243	-
GcEpN	1.23	0.5	1.5	7.449	0.002974	0.000399	-
GcEpD	0.74	0.5	1.5	7.357	0.1376	0.01871	-
GintN	1	0.5	1.5	7.447	4.36E-05	5.85E-06	-
GintD	1	0.5	1.5	7.449	0.01635	0.002195	-
GimIntN	1.23	0.5	1.5	7.447	4.11E-05	5.52E-06	-
GimIntD	0.74	0.5	1.5	7.446	0.001797	0.000241	-
GsmN	1.23	0.5	1.5	7.461	0.01669	0.002237	-
GsmD	0.74	0.5	1.5	6.921	0.7033	0.1016	-
GcEdN	1.23	0.5	1.5	7.447	0.000171	2.30E-05	-
GcEdD	0.74	0.5	1.5	7.441	0.006594	0.000886	-
GpN	1	0.5	1.5	7.447	8.91E-09	1.20E-09	-

GpD	1	0.5	1.5	7.447	1.99E-08	2.67E-09	-
AaEp	1.08E-02	2.70E-03	4.32E-02	7.291	3.757	0.5153	-
AimInt	1.08E+04	1.08E-05	1.08E-03	7.447	8.62E-08	1.16E-08	+
Asm	2.16E-02	2.16E-03	2.16E-01	15.18	4.173	0.2749	+
AbEd	2.16E-03	5.40E-04	8.64E-03	7.152	1.164	0.1627	-
AaEd	2.16E-03	5.40E-04	8.64E-03	7.116	1.149	0.1614	-
ASL	15	1.5	150	4.889	1.849	0.3781	-
VcEp	7.20E-08	7.20E-09	7.20E-07	5.656	1.082	0.1912	-
Vint	1.08E-08	1.08E-09	1.08E-07	6.774	0.4348	0.06419	-
Vsm	4.70E-08	4.70E-09	4.70E-07	15.56	4.529	0.2911	+
pHaEp	7.4	4	9	7.448	0.1056	0.01418	-
pHcEp	7.0	4	9	7.409	0.07615	0.01028	-
pHint	7.0	4	9	7.805	0.6839	0.08762	+
pHsm	7.0	4	9	7.309	2.272	0.3109	-
pHimInt	7.0	4	9	7.446	0.002912	0.000391	-
pHcEd	7.0	4	9	7.445	0.02066	0.002775	-
pHp	7.4	4	9	7.447	3.19E-08	4.28E-09	-
EbEd	-0.06	-0.12	0	8.59	0.5854	0.06815	+
EaEd	-0.06	-0.12	0	7.79	0.2234	0.02868	-
Esm	-0.06	-0.12	0	10.46	6.49	0.6205	-
EimInt	-0.06	-0.12	0	7.449	0.007372	0.00099	-
EbEp	0.0119	0	0.12	6.296	0.6186	0.09824	-
EaEp	-0.0093	-0.12	0	6.958	0.3514	0.05051	+
Ro	0.1	0.01	0.5	10.44	2.555	0.2448	+

Table. S4 Parameter values and sensitivity analysis of T_{ss} (min) for alveoli

Parameter	Default	low	high	Mean	SD	CV	Trend
LaEp	0.95	0.05	0.95	1.795	0.6543	0.3645	+
LimEp	0.05	0.05	0.5	3.297	0.1911	0.05796	+
LcEp	0.05	0.005	0.5	3.021	0.01608	0.005323	+
Lint	0	0.005	0.5	2.992	0.006055	0.002024	-
LimInt	0.05	0.005	0.5	3.016	0.01673	0.005546	+
LcEd	0.05	0.005	0.5	2.912	0.05801	0.01992	-
Lp	0	0.005	0.5	2.998	0.004398	0.001467	-
GaEpN	1	0.5	1.5	3.038	0.1957	0.06441	-
GaEpD	1	0.5	1.5	3.335	0.8703	0.261	-
GimEpN	1.23	0.5	1.5	3.002	0.002848	0.000949	-
GimEpD	0.74	0.5	1.5	2.97	0.05358	0.01804	-
GcEpN	1.23	0.5	1.5	3.001	0.001427	0.000476	+
GcEpD	0.74	0.5	1.5	2.994	0.007105	0.002373	-
GintN	1	0.5	1.5	3.001	0.001191	0.000397	+
GintD	1	0.5	1.5	2.997	0.004986	0.001664	+
GimIntN	1.23	0.5	1.5	2.999	0.002187	0.000729	+
GimIntD	0.74	0.5	1.5	2.998	0.004614	0.001539	-
GcEdN	1.23	0.5	1.5	2.998	0.004602	0.001535	+

GcEdD	0.74	0.5	1.5	3.024	0.03149	0.01041	+
GpN	1	0.5	1.5	3.001	0.00178	0.000593	-
GpD	1	0.5	1.5	2.998	0.004921	0.001641	-
AaEp	0.387	0.0387	3.87	3.259	0.725	0.2225	+
AbEp	0.387	0.0387	3.87	2.924	0.3595	0.1229	-
AimEp	0.0042	0.00042	0.042	2.956	0.03784	0.0128	-
AimInt	0.00042	4.19E-05	0.004192	2.997	0.009786	0.003266	-
AbEd	0.452	0.0452	4.52	2.7	0.2743	0.1016	-
AaEd	0.452	0.0452	4.52	2.774	0.3023	0.109	-
ASL	5	0.5	50	15.3	9.311	0.6087	+
Vint	2.68E-07	2.68E-08	2.68E-06	2.983	0.009908	0.003322	-
EaEp	-0.0093	-0.12	0	1.162	0.08901	0.07661	+
EbEp	0.0119	0	0.12	1.651	0.0762	0.04614	-
EimEp	-0.06	-0.12	0	3.147	0.293	0.09308	-
EimInt	-0.06	-0.12	0	3.007	0.01809	0.006017	-
EbEd	-0.06	-0.12	0	3.338	1.073	0.3214	+
EaEd	-0.06	-0.12	0	3.119	0.3052	0.09785	-
pHaEp	7.4	4	9	2.998	0.09591	0.03199	+
pHimEp	7	4	9	2.977	0.08239	0.02767	-
pHcEp	7	4	9	3.138	0.2374	0.07565	+
pHint	7	4	9	3.226	0.3662	0.1135	+
pHimInt	7	4	9	2.998	0.004545	0.001516	-
pHcEd	7	4	9	3.063	0.1185	0.03868	+
pHp	7.4	4	9	2.997	0.005152	0.001719	-
Ro	0.1	0.01	0.5	2.921	0.06519	0.02232	-

Table. S5 Parameter values and sensitivity analysis of T_{ss} (min) for airways

Parameter	default	low	high	Mean	SD	CV	Trend
LaEp	0.2	0.05	0.95	34.37	3.208	0.09334	+
LcEp	0.05	0.005	0.5	32.61	1.097	0.03365	+
Lint	0	0.005	0.5	31.26	0.1733	0.005542	+
LimInt	0.05	0.005	0.5	30.99	0.05303	0.001711	-
Lsm	0.05	0.005	0.5	41.89	7.162	0.171	+
LcEd	0.05	0.005	0.5	31	0.05826	0.001879	-
Lp	0	0.005	0.5	30.96	0.0523	0.001689	+
GaEpN	1	0.5	1.5	31	0.1873	0.006043	-
GaEpD	1	0.5	1.5	31.48	1.314	0.04172	-
GcEpN	1.23	0.5	1.5	30.93	0.03826	0.001237	+
GcEpD	0.74	0.5	1.5	30.65	0.4926	0.01607	-
GintN	1	0.5	1.5	30.93	0.02438	0.000788	-
GintD	1	0.5	1.5	30.99	0.09294	0.002999	-
GimIntN	1.23	0.5	1.5	30.92	0.03475	0.001124	+
GimIntD	0.74	0.5	1.5	30.97	0.04729	0.001527	-

GsmN	1.23	0.5	1.5	31.03	0.1006	0.003242	-
GsmD	0.74	0.5	1.5	28.41	3.055	0.1075	-
GcEdN	1.23	0.5	1.5	30.95	0.04532	0.001465	-
GcEdD	0.74	0.5	1.5	30.95	0.04737	0.00153	+
GpN	1	0.5	1.5	30.93	0.02644	0.000855	+
GpD	1	0.5	1.5	30.97	0.05493	0.001774	-
AaEp	1.08E-02	2.70E-03	4.32E-02	31.08	10.23	0.3292	-
AimInt	1.08E+04	1.08E-05	1.08E-03	30.96	0.04623	0.001493	-
Asm	2.16E-02	2.16E-03	2.16E-01	124.4	66.76	0.5367	-
AbEd	2.16E-03	5.40E-04	8.64E-03	22.11	9.556	0.4322	-
AaEd	2.16E-03	5.40E-04	8.64E-03	22.8	10.32	0.4529	-
ASL	15	1.5	150	50.52	15.21	0.301	+
VcEp	7.20E-08	7.20E-09	7.20E-07	45.77	10.24	0.2237	+
Vint	1.08E-08	1.08E-09	1.08E-07	32.05	0.7181	0.02241	+
Vsm	4.70E-08	4.70E-09	4.70E-07	124.4	66.76	0.5367	+
EaEp	-0.0093	-0.12	0	28.33	1.249	0.04409	+
EbEp	0.0119	0	0.12	26.38	2.277	0.08634	-
Esm	-0.06	-0.12	0	40.18	26.64	0.6632	-
EimInt	-0.06	-0.12	0	30.99	0.06302	0.002034	-
EbEd	-0.06	-0.12	0	47.79	3.545	0.07418	+
EaEd	-0.06	-0.12	0	34.99	1.05	0.03001	-
pHaEp	7.4	4	9	30.98	0.2709	0.008745	-
pHcEp	7	4	9	30.79	0.2611	0.008477	-
pHint	7	4	9	32.87	3.068	0.09333	+
pHimInt	7	4	9	30.95	0.05372	0.001736	+

pHcEd	7	4	9	30.94	0.05553	0.001795	-
pHp	7.4	4	9	30.97	0.06052	0.001954	+
Ro	0.1	0.01	0.5	51.29	17.51	0.3415	+

Table. S6 Parameter values and sensitivity analysis of mass deposition for alveoli (mass fraction in lungs)

Parameter	Default	low	high	Mean	SD	CV	Trend
LaEp	0.95	0.05	0.95	0.8867	0.02451	0.02764	+
LimEp	0.05	0.005	0.5	0.9215	0.000416	0.000452	+
LcEp	0.05	0.005	0.5	0.9215	0.000432	0.000469	+
Lint	0	0.005	0.5	0.9222	0.001027	0.001114	+
LimInt	0.05	0.005	0.5	0.921	9.63E-05	0.000105	+
LcEd	0.05	0.005	0.5	0.9276	0.004776	0.005148	+

Lp	0	0.005	0.5	0.8295	0.05402	0.06512	-
GaEpN	1	0.5	1.5	0.9153	0.003549	0.003877	-
GaEpD	1	0.5	1.5	0.9181	0.01396	0.01521	-
GimEpN	1.23	0.5	1.5	0.9147	4.31E-06	4.71E-06	-
GimEpD	0.74	0.5	1.5	0.9146	0.000202	0.00022	-
GcEpN	1.23	0.5	1.5	0.9147	4.96E-06	5.43E-06	-
GcEpD	0.74	0.5	1.5	0.9146	0.000211	0.000231	-
GintN	1	0.5	1.5	0.9147	1.22E-05	1.34E-05	-
GintD	1	0.5	1.5	0.9149	0.000565	0.000618	-
GimIntN	1.23	0.5	1.5	0.9147	9.48E-07	1.04E-06	-
GimIntD	0.74	0.5	1.5	0.9147	3.95E-05	4.32E-05	-
GcEdN	1.23	0.5	1.5	0.9148	6.31E-05	6.89E-05	-
GcEdD	0.74	0.5	1.5	0.9133	0.002762	0.003024	-
GpN	1	0.5	1.5	0.9147	1.79E-09	1.95E-09	+
GpD	1	0.5	1.5	0.9097	0.02491	0.02738	+
AaEp	0.387	0.0387	3.87	0.9279	0.02019	0.02176	+
AbEp	0.387	0.0387	3.87	0.9208	2.48E-15	2.70E-15	-
AimEp	0.0042	0.00042	0.042	0.9242	0.002247	0.002431	+

AimInt	0.00042	4.19E-05	0.004192	0.9208	2.61E-15	2.83E-15	-
AbEd	0.452	0.0452	4.52	0.9511	0.01806	0.01899	+
AaEd	0.452	0.0452	4.52	0.9208	2.30E-15	2.50E-15	+
ASL	5	0.5	50	0.9693	0.02505	0.02584	+
Vint	2.68E-07	2.68E-08	2.68E-06	0.9266	0.004073	0.004395	+
EaEp	-0.0093	-0.12	0	0.7143	0.1568	0.2196	+
EbEp	0.0119	0	0.12	0.8432	0.05729	0.06795	-
EimEp	-0.06	-0.12	0	0.921	0.00079	0.000858	-
EimInt	-0.06	-0.12	0	0.9209	0.000192	0.000209	-
EbEd	-0.06	-0.12	0	0.9152	0.05026	0.05491	+
EaEd	-0.06	-0.12	0	0.8826	0.09135	0.1035	-
pHaEp	7.4	4	9	0.9213	0.002439	0.002647	+
pHimEp	7	4	9	0.9207	0.000335	0.000364	-
pHcEp	7	4	9	0.9246	0.006427	0.006951	+
pHint	7	4	9	0.9279	0.01363	0.01469	+
pHimInt	7	4	9	0.9208	6.61E-05	7.18E-05	-
pHcEd	7	4	9	0.9169	0.008666	0.009452	-
pHp	7.4	4	9	0.9284	0.01869	0.02013	+

Ro	0.1	0.01	0.5	0.9302	0.008617	0.009263	+

Table. S7 Parameter and sensitivity analysis of mass deposition for airways (mass fraction in lungs)

Paramet er	Default	low	high	Mean	SD	CV	Trend
LaEp	0.2	0.05	0.95	0.08661	0.006106	0.0705	+
LcEp	0.05	0.005	0.5	0.08279	0.002397	0.02896	+
Lint	0	0.005	0.5	0.07976	0.000444	0.00556	+
Lsm	0.05	0.005	0.5	0.1035	0.01537	0.1485	+
LimInt	0.05	0.005	0.5	0.07923	3.87E-05	0.000489	+
LcEd	0.05	0.005	0.5	0.07966	0.000349	0.004382	+
Lp	0	0.005	0.5	0.03946	0.001595	0.04042	-
GaEpN	1	0.5	1.5	0.07924	0.000363	0.004582	-
GaEpD	1	0.5	1.5	0.08016	0.002608	0.03254	-

GcEpN	1.23	0.5	1.5	0.07919	2.40E-05	0.000303	-
GcEpD	0.74	0.5	1.5	0.07833	0.001	0.01277	-
GintN	1	0.5	1.5	0.07917	6.29E-06	7.94E-05	-
GintD	1	0.5	1.5	0.07929	0.00027	0.003408	-
GsmN	1.23	0.5	1.5	0.07936	0.000182	0.002295	-
GsmD	0.74	0.5	1.5	0.07293	0.007359	0.1009	-
GimIntN	1.23	0.5	1.5	0.07917	4.28E-07	5.40E-06	-
GimIntD	0.74	0.5	1.5	0.07916	1.75E-05	0.000221	-
GcEdN	1.23	0.5	1.5	0.07917	3.46E-06	4.36E-05	-
GcEdD	0.74	0.5	1.5	0.07904	0.00013	0.00165	-
GpN	1	0.5	1.5	0.07913	0.000155	0.001964	+
GpD	1	0.5	1.5	0.07391	0.002036	0.02755	+
AaEp	1.08E-02	2.70E-03	4.32E-02	0.08063	0.00408	0.0506	+
AimInt	1.08E+04	1.08E-05	1.08E-03	0.07945	0.000201	0.002525	+
Asm	2.16E-02	2.16E-03	2.16E-01	0.2206	0.1051	0.4766	+
AbEd	2.16E-03	5.40E-04	8.64E-03	0.07947	0.000445	0.005597	+
AaEd	2.16E-03	5.40E-04	8.64E-03	0.0833	0.002652	0.03184	+
Vint	1.08E-08	1.08E-09	1.08E-07	0.08255	0.00229	0.02774	+
EaEp	-0.0093	-0.12	0	0.07357	0.002812	0.03823	+
EbEp	0.0119	0	0.12	0.0688	0.005865	0.08524	-
Esm	-0.06	-0.12	0	0.09275	0.06086	0.6562	-
EimInt	-0.06	-0.12	0	0.0792	8.91E-05	0.001125	-
EbEd	-0.06	-0.12	0	0.1321	0.01128	0.08541	+
EaEd	-0.06	-0.12	0	0.0943	0.007571	0.08028	-
pHaEp	7.4	4	9	0.07907	0.00068	0.008595	-

pHcEp	7	4	9	0.07879	0.000626	0.007948	-
pHint	7	4	9	0.08395	0.007481	0.08911	+
pHsm	7	4	9	0.07023	0.02485	0.3538	-
pHimInt	7	4	9	0.07916	3.29E-05	0.000416	-
pHcEd	7	4	9	0.07901	0.000458	0.005797	-
pHp	7.4	4	9	0.09632	0.003901	0.0405	+
Ro	0.1	0.01	0.5	0.119	0.03917	0.3292	+
